# Structural basis for translation inhibition by the glycosylated drosocin peptide

**DOI:** 10.1038/s41589-023-01293-7

**Published:** 2023-03-30

**Authors:** Timm O. Koller, Martino Morici, Max Berger, Haaris A. Safdari, Deepti S. Lele, Bertrand Beckert, Kanwal J. Kaur, Daniel N. Wilson

**Affiliations:** 1 Institute for Biochemistry and Molecular Biology, University of Hamburg, Hamburg, Germany; 2 National Institute of Immunology, Aruna Asaf Ali Marg, New Delhi, India; 3 Dubochet Center for Imaging (DCI) at EPFL, EPFL SB IPHYS DCI, Lausanne, Switzerland

**Keywords:** Mechanism of action, Translation, RNA

## Abstract

The proline-rich antimicrobial peptide (PrAMP) drosocin is produced by *Drosophila* species to combat bacterial infection. Unlike many PrAMPs, drosocin is O-glycosylated at threonine 11, a post-translation modification that enhances its antimicrobial activity. Here we demonstrate that the O-glycosylation not only influences cellular uptake of the peptide but also interacts with its intracellular target, the ribosome. Cryogenic electron microscopy structures of glycosylated drosocin on the ribosome at 2.0–2.8-Å resolution reveal that the peptide interferes with translation termination by binding within the polypeptide exit tunnel and trapping RF1 on the ribosome, reminiscent of that reported for the PrAMP apidaecin. The glycosylation of drosocin enables multiple interactions with U2609 of the 23S rRNA, leading to conformational changes that break the canonical base pair with A752. Collectively, our study reveals novel molecular insights into the interaction of O-glycosylated drosocin with the ribosome, which provide a structural basis for future development of this class of antimicrobials.

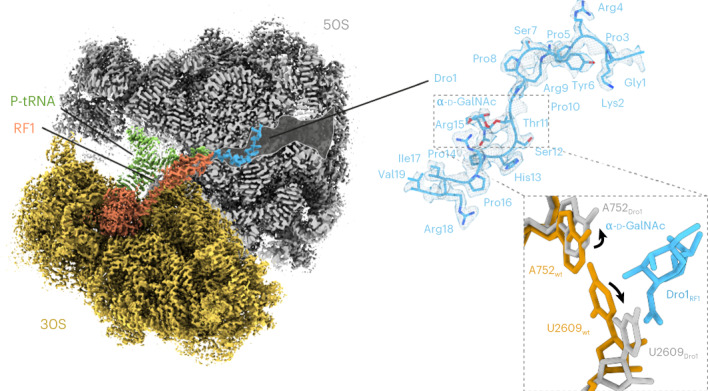

## Abstract (web summary)

Koller et al. determined structures of the O-glycosylated antimicrobial peptide drosocin from *Drosophila melanogaster* in complex with the bacterial ribosome, revealing the mechanism by which drosocin inhibits the termination phase of protein synthesis.

## Main

The host defense systems of mammals and higher insects produce a battery of potent antimicrobial peptides (AMPs) in response to bacterial infection. Unlike most AMPs that kill bacteria using a lytic mechanism, proline-rich AMPs (PrAMPs) pass through the bacterial membrane and target intracellular processes, such as protein synthesis^1–3^. Two types of PrAMPs have been identified and classified based on their mechanism of action to inhibit protein synthesis—namely, type I PrAMPs that block the accommodation of the aminoacyl-tRNA directly after translation initiation and type II PrAMPs that do not interfere with initiation and elongation but prevent dissociation of the release factors RF1 and RF2 during the termination phase^3^. Structures on the ribosome of a variety of type I PrAMPs from both insect (oncocin, metalnikowin I and pyrrhocoricin) and mammalian (Bac7 and Tur1A) origin have revealed overlapping binding sites that span from the ribosomal exit tunnel to the A-site of the peptidyltransferase center (PTC)^4–9^. It has been proposed that, by occluding the A-site at the PTC on the ribosome, type I PrAMPs prevent the binding of the aminoacylated CCA-end of the incoming A-site tRNA and, thereby, arrest translation^3–7^. Structures on the ribosome with the type II PrAMP Api137, a synthetic derivative of the natural PrAMP apidaecin, have revealed a binding site within the ribosomal exit tunnel that overlaps with type I PrAMPs^10,11^. However, the binding mode of Api137 is completely different, with a reversed orientation compared to type I PrAMPs, and also Api137 does not encroach so markedly on the A-site of the PTC. Instead, Api137 inhibits translation by trapping the termination release factors on the ribosome after peptidyl-tRNA hydrolysis^10,11^.

**Fig. 1:**
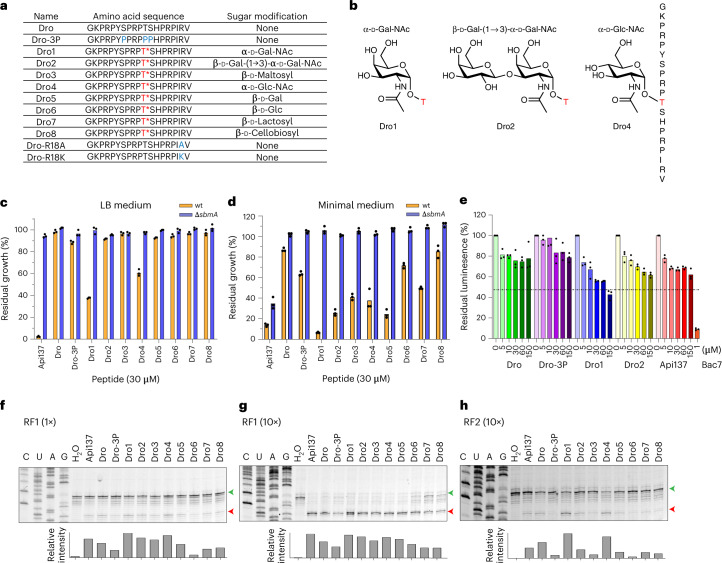
Characterization of inhibitory activity of drosocin derivatives. **a**, Amino acid sequences of the drosocin peptides used in this study. Drosocin peptides carrying a modification on Thr11 are indicated with T*, whereas the mutated positions are shown in blue. **b**, Chemical structures of the Thr11 modifications of Dro1, Dro2 and Dro4. **c**,**d**, In vivo inhibitory activity of 30 μM Api137 and drosocin derivatives on the growth of *E. coli* wild-type (yellow) and Δ*sbmA* (blue) strains in rich LB (**c**) or minimal medium (**d**). Histograms represent the averages from three biological replicates, individually plotted as dots, and results are normalized to growth in the absence of peptide, which was assigned as 100%. **e**, Inhibitory activity of increasing concentrations of Dro (green), Dro-3P (purple), Dro1 (blue), Dro2 (yellow), Api137 (red) and 1 μM Bac7 (orange) on in vitro translation using Fluc as a reporter. The luminescence in the absence of compounds was normalized to 100%; experiments were performed in triplicate, individually plotted as dots, and the bars represent the mean. **f**–**h**, Toeprinting assays monitoring the position of ribosomes on an MLIF*-mRNA in the presence of 30 μM Api137 and drosocin derivatives and 1× RF1 (**f**), 10× RF1 (**g**) or 10× RF2 (**h**). Bands corresponding to ribosomes present at the start and stop codons are indicated by green and red arrows, respectively. The histogram represents the proportion of relative intensity of stop codon band for the different peptides. Toeprinting assays were performed in duplicate. wt, wild-type. Source data

In addition to the classical membrane-targeting AMPs, such as defensins, cecropins and diptericins, *Drosophila* also produce a PrAMP called drosocin^12,13^. Drosocin is 19 amino acids long, rich in proline and arginine residues^12^ (Fig. 1a) and displays excellent activity against Gram-negative bacteria, such as *Escherichia coli*^12,13^. However, unlike most PrAMPs, drosocin carries an O-glycosylation on residue Thr11, consisting of either the monosaccharide N-acetylgalactosamine (α-d-GalNAc) or a disaccharide comprising galactose linked to an N-acetylgalactosamine (β-Gal(1→3)-α-d-GalNAc) (Fig. 1a,b)^12,14^. A double-glycosylated form of drosocin bearing the monosaccharide on Ser7 as well as Thr11 has also been reported^15^. Both the monosaccharide and disaccharide forms of drosocin appear in *Drosophila* hemolymph within 6 hours after infection and increase in concentration (to 40 μM) for up to 24 hours^14^. Although the disaccharide form disappears 2 weeks after infection, the monosaccharide persists for up to 3 weeks^14^. Synthetic drosocin lacking O-glycosylation is less active than the native compounds, suggesting that the post-translational modification is necessary for full activity^12,13,16,17^. Indeed, many studies have demonstrated that a variety of synthetic drosocin derivatives with varying sugar moieties maintain good antimicrobial activity, generally better than the unmodified form^17–24^. Although nuclear magnetic resonance and circular dichroism experiments suggest that both the modified and unmodified forms of drosocin adopt extended conformations in solution^17,23,24^, the presence of the modification has nevertheless been proposed to help drosocin maintain an extended conformation to facilitate binding to its intracellular target^13,17^. Additionally, glycosylation can also increase solubility and serum stability and broaden the biological activity spectrum^13^; however, the exact role of glycosylation for drosocin is unknown.

Although drosocin inhibits protein synthesis in vivo and in vitro^25,26^, the exact mechanism remains unclear. Interestingly, the type I insect PrAMP pyrrhocoricin is O-glycosylated with N-acetylgalactosamine on Thr11, and a minor disaccharide form with the additional galactose has also been detected^27^. Together with the reported sequence similarity, drosocin was proposed to act analogously to pyrrhocoricin and metalnikowins rather than apidaecins and abaecins^13^. However, several subsequent observations support similarity between drosocin and apidaecin rather than type I PrAMPs. First, in contrast to drosocin, unmodified pyrrhocoricin is slightly more active than the modified form^16^. Second, drosocin was suggested to belong to the apidaecin-like PrAMPs based on similarity in terms of ribosome-binding antibiotic competition assays—that is, drosocin competes better with Api137 than with the oncocin derivative Onc112 (ref. ^28^). Finally, drosocin lacking the carboxy-terminal Arg18–Val19 almost completely loses antimicrobial activity^16^, analogous to Api137 (ref. ^29^), whereas N-terminal, rather than C-terminal, truncations inactivate type I PrAMPs, such as Bac7 (refs. ^6,30^).

In this study, we employed biochemical and structural approaches to dissect the mechanism by which drosocin interacts with the ribosome and inhibits protein synthesis, and we shed light on the role of the critical O-glycosylation on Thr11.

## Results

### Characterization of the activity of drosocin derivatives

Many PrAMPs use the SbmA transporter to pass through the *E. coli* inner membrane^4,10,31^; however, whether drosocin also uses SbmA remains, to our knowledge, unknown. To address this, we monitored the effect of the presence of diverse drosocin peptides (Fig. 1a,b and Supplementary Fig. 1a) on the growth of the wild-type *E. coli* strain BW25113 containing SbmA or lacking SbmA (Δ*sbmA*) (Fig. 1c,d). We compared unmodified drosocin (Dro) with various modified forms (Fig. 1a,b). The modified forms included the naturally occurring Dro1 and Dro2 that carry either a monosaccharide (α-d-GalNAc) or disaccharide (β-d-Gal(1→3)-α-d-GalNAc) attached to Thr11, respectively (Fig. 1b). In addition, we examined the previously reported^23,24^ drosocin derivatives bearing β-d-Maltosyl (Dro3), α-d-GlcNAc (Dro4), β-d-Gal (Dro5), β-d-Glc (Dro6), β-d-Lactosyl (Dro7) and β-d-Cellobiosyl (Dro8) modifications on Thr11 (Fig. 1a,b and Supplementary Fig. 1a). Finally, we also included in our analysis the synthetic unmodified drosocin derivative with proline substitutions at positions 7, 11 and 12 (Dro-3P) (Fig. 1a), which was previously reported to have similar antimicrobial activity to the monosaccharide form of drosocin^25^. Growth was monitored in both rich (LB) and minimal medium in the presence of 30 μM of each peptide and normalized with the growth in the absence of the compounds (Methods). In rich medium, we observed growth inhibition only with Dro1 and Dro4 (Fig. 1c). Because no inhibition was observed with the monosaccharide β-d-Gal (Dro5) or β-d-Glc (Dro6) drosocins, this suggests that, under these conditions, the stereochemistry of the anomeric carbon on the sugar is more important than the type of sugar itself. We also observed no inhibition for Dro2 nor for any of the β-linked disaccharides (Dro3, Dro6 or Dro7). Similarly, the unmodified drosocin and Dro-3P variant were inactive under these conditions (Fig. 1c). By contrast, all drosocin peptides inhibited growth of the *E. coli* BW25113 strain in minimal medium, albeit to different extents (Fig. 1d). The trends were similar to that reported previously^23,24^—namely, with the highest inhibition observed using Dro1 and the lowest with the unmodified peptide—whereas the other glycosylated variants lay in between (Fig. 1d). We did not observe similar activity between Dro1 and Dro-3P, as reported previously^25^, which may arise due to differences in the *E. coli* strains and/or growth conditions used. Strikingly, we note that any inhibition observed with the *E. coli* BW25113 strain was lost when performed with the BW25113 Δ*sbmA* strain, indicating that SbmA plays a major role in the cellular uptake of all drosocin peptides.

### Drosocin traps ribosomes on stop codons during translation

Unmodified wild-type drosocin and Dro-3P have been reported to inhibit in vitro translation reactions^25,26^; however, the naturally occurring glycosylated forms of drosocin have not been previously tested. To investigate this, we compared the effect of increasing concentrations (0–150 μM) of modified Dro1 and Dro2 with unmodified Dro, Dro-3P and Api137 using a cell-free in vitro translation system and firefly luciferase (Fluc) mRNA as a template (Fig. 1e), as we have used previously for assessing the activity of other PrAMPs^4,6,8,9,32,33^. Dro1 exhibited dose-dependent inhibition, with a half maximal inhibitory concentration (IC_50_) of 78 ± 13 μM and reaching a maximum of 60% inhibition at the highest concentration tested of 150 μM. By contrast, both Dro and Dro-3P were poor inhibitors, reaching a maximum of 20% inhibition at 150 μM, whereas Dro2 and Api137 were slightly more effective, with 40% inhibition observed at 150 μM. This contrasts with type I PrAMPs, such as Bac7 (Fig. 1e) and Onc112, that display IC_50_ of <1 μM using the same system^4,6^, suggesting that drosocin may inhibit translation similarly to Api137, as proposed previously^28^.

To ascertain which step during protein synthesis is affected by drosocin, we performed toeprinting assays, where reverse transcription is used to monitor the position of ribosomes on a defined mRNA^34^. In the absence of PrAMP, but the presence of RF1, we observed no band corresponding to ribosomes at the UAA stop codon of the mRNA, whereas, in the presence of 25 μM Api137 and RF1, ribosomes become stuck at the stop codon (Fig. 1f and Supplementary Fig. 1b), as expected^10^. Similarly, the same termination band was also observed in the presence of 30 μM of each of the tested drosocin derivatives, albeit with differing intensities (Fig. 1f). Increasing the concentration of RF1 by ten-fold in the reactions led to more intense termination bands (Fig. 1g and Supplementary Fig. 1c), consistent with a role of drosocin acting during the termination phase, as reported for Api137 (refs. ^10,11^). We performed the same toeprinting reactions in the presence of ten-fold RF2, rather than ten-fold RF1, and also observed stalling of ribosomes at the stop codon, albeit with much lower efficiency (Fig. 1h and Supplementary Fig. 1d). The weaker stalling with RF2 is likely due to the endemic A246T mutation found in RF2 from K12 strains, which likely confers some resistance to drosocin, as was shown previously for Api137 (ref. ^10^). The strongest stalling was observed in the presence of Dro1 and, to a lesser extent, with Dro4, a trend that was particularly evident in the presence of ten-fold RF2 (Fig. 1h). Both Dro1 and Dro4 were also the most active in our whole-cell assays (Fig. 1c,d). By contrast, weak stalling was observed with Dro-3P, consistent with the lack of activity in the whole-cell (Fig. 1c,d) and in vitro translation assays (Fig. 1e). Interestingly, we observed good activity for Dro in the toeprinting assay (Fig. 1f–h), suggesting that the poor activity observed in the whole-cell assays (Fig. 1c, d) may be due to cellular uptake.

### Cryogenic electron microscopy structures of drosocin-bound ribosome complexes

**Fig. 2:**
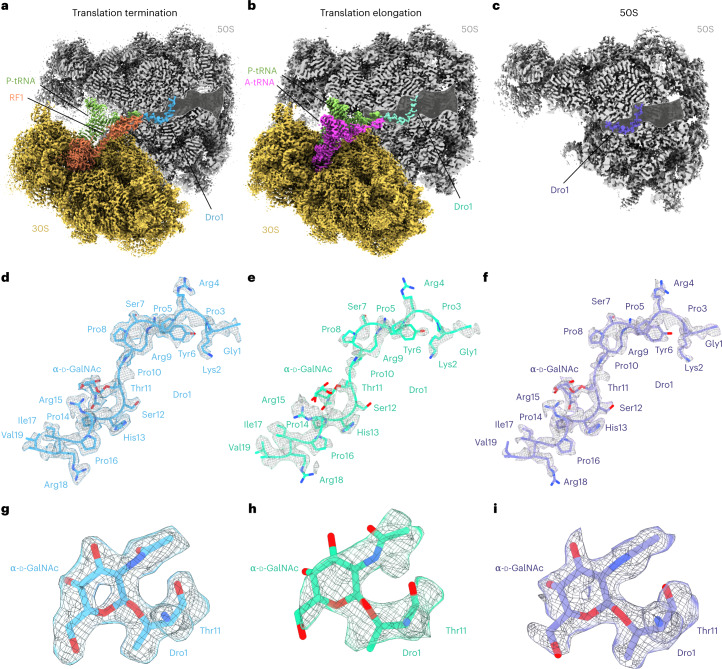
Cryo-EM structures of drosocin-bound ribosomal complexes. **a**–**c**, Cryo-EM maps of Dro1 bound to termination (**a**) and elongation (**b**) complexes as well as the large 50S subunit (**c**), with transverse section of the 50S (gray) to reveal the Dro1 binding site within the exit tunnel. In **a**, the P-tRNA, RF1 and Dro1 are colored green, orange and cyan, respectively. In **b**, the A-tRNA, P-tRNA and Dro1 are colored pink, green and teal, respectively, whereas, in **c**, Dro1 is purple. **d**–**f**, Cryo-EM density (gray mesh) with molecular model for Dro1 (cyan) (**d**) from termination complex as in **a**, Dro1 (teal) (**e**) from elongation complex as in **b** and Dro1 (purple) (**f**) from the 50S subunit as in **c**. **g**–**i**, Cryo-EM density (gray mesh) with molecular model for α-d-GalNAc modification at Thr11 of Dro1 in the termination (**g**) and elongation (**h**) complexes as well as the 50S subunit (**i**).

**Extended Data Fig. 1:**
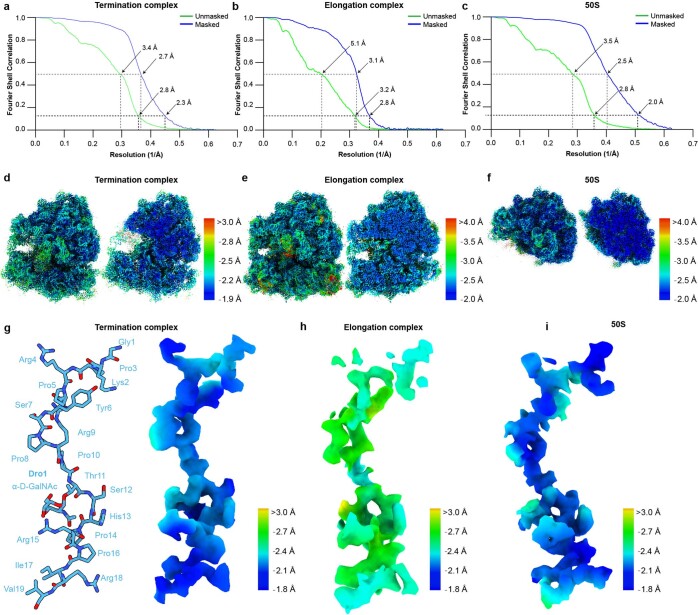
Cryo-EM data processing. **a-c**, Fourier shell correlation (FSC) curve of the (**a**) termination, (**b**) elongation and (**c**) 50S complexes, with unmasked (green) and masked (blue) FSC curves plotted against the resolution (1/Å). **d-f**, Cryo-EM density colored according to local resolution and transverse section for the (**d**) termination, (**e**) elongation and (**f**) 50S complexes. **g-i**, Molecular model of Dro1 (light blue) and corresponding cryo-EM density colored according to local resolution for the (**g**) termination, (**h**) elongation and (**i**) 50S complex.

**Extended Data Fig. 2:**
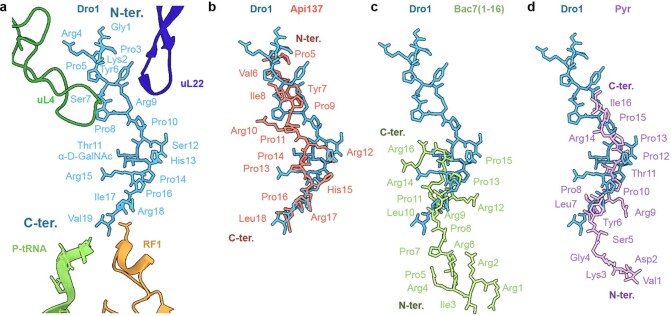
Dro1 binds with same orientation as Api137, which is distinct from Bac7 and Pyr. **a**, Relative position of Dro1 (light blue) compared to P-tRNA (lime), RF1 (orange), uL4 (green) and uL22 (dark blue) within the Dro1-bound termination complex. **b-d**, Superimposition of Dro1 (light blue) from **(a)** with **(b)** Api137 (salmon) from the Api137-ArfB complex (PDB ID 6YSS)^61^, **(c)** Bac7(1–16) (lime) from the Bac7-70S complex (PDB ID 5F8K)^6^ and **(d)** Pyrrhocoricin (Pyr, purple) from the Pyr-70S complex (PDB ID 5FDV)^6^.

To investigate how drosocin inhibits translation and to provide insight into the role of the O-glycosylation, we set out to determine a cryogenic electron microscopy (cryo-EM) structure of a ribosome–drosocin complex. Rather than forming complexes with vacant ribosomes or pre-defined functional states, we instead performed translation reactions with the same mRNA template used for the toeprinting assays in the presence of ten-fold RF1 and 30 μM Dro1 (Fig. 1g). Reactions were subsequently pelleted through sucrose cushions, and the pelleted ribosomal complexes were subjected to single-particle cryo-EM analysis. In silico sorting of the data revealed three main populations of ribosomal states—namely, 70S ribosomes with RF1 and P-site tRNA (26%) or with A-site and P-site tRNAs (16%) as well as a population containing only large 50S subunits (30%) (Supplementary Fig. 2), which, after refinement, yielded final reconstructions at 2.3 Å, 2.8 Å and 2.0 Å, respectively (Fig. 2a–c and Extended Data Fig. 1). In all three reconstructions, additional density was observed within the ribosomal exit tunnel that could be unambiguously assigned to the drosocin peptide (Fig. 2a–i). The density for drosocin was particularly well resolved in the RF1-containing 70S map enabling all 19 amino acids to be modeled with sidechains (Fig. 2d and Extended Data Fig. 1), including the α-d-GalNAc modification linked to Thr11 (Fig. 2g). Similarly, the density for drosocin in the cryo-EM map of the 50S subunit was generally well resolved, except for the N-terminal and C-terminal regions (Fig. 2f,i and Extended Data Fig. 1). By contrast, the density for drosocin in the cryo-EM map of the complex containing A-site and P-site tRNAs was less well resolved (Fig. 2e and Extended Data Fig. 1) and was particularly poor for the α-d-GalNAc modification (Fig. 2h), suggesting that the peptide is bound less stably within this complex. Nevertheless, in all three structures, the overall orientation of the drosocin peptide within the exit tunnel was identical, with the C-terminus located at the PTC and the N-terminus extending into the exit tunnel, analogous to an elongating nascent polypeptide chain (Fig. 2a–f and Extended Data Fig. 2a). This orientation is also the same as that observed for the type II PrAMP Api137 (refs. ^10,11^) but opposite to that of type I PrAMPs, such as Bac7 and pyrrhocoricin (Extended Data Fig. 2b–d)^4–9^.

### Cryo-EM structure of drosocin on an elongating ribosome

**Fig. 3:**
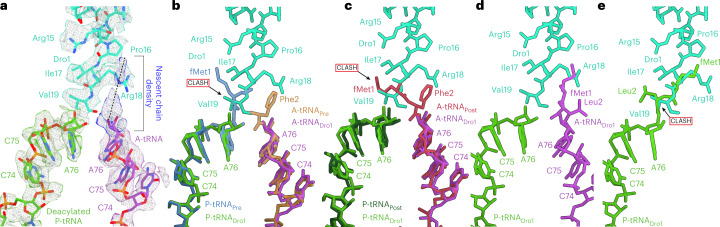
Cryo-EM structure of the drosocin-bound translation elongation complex. **a**, Isolated cryo-EM densities (mesh) with molecular models for P-tRNA (light green), A-tRNA (magenta) and Dro1 (teal) within the translation elongation complex. Additional density connected to the A-site tRNA is attributed to the nascent chain but cannot be modeled due to flexibility. **b**, Superimposition of P-tRNA_Dro1_ (light green), A-tRNA_Dro1_ (purple) and Dro1 (teal) from **a**, with P-tRNA_Pre_ (blue) and A-tRNA_Post_ (brown) from PRE-state (PDB ID: 1VY4)^35^. Alignment is based on the 23S rRNA. The fMet attached to the P-site tRNA would be predicted to clash with the C-terminus of Dro1 (teal). **c**, Superimposition of P-tRNA_Dro1_ (light green), A-tRNA_Dro1_ (purple) and Dro1 (teal) from **a**, with P-tRNA_Post_ (dark green) and A-tRNA_Post_ (red) from POST-state (PDB ID: 1VY5)^35^. Alignment is based on the 23S rRNA. The fMet from the fMet-Phe, attached to the A-tRNA_Post_, would be predicted to clash with the C-terminus of Dro1 (teal). **d**, Hypothetical molecular model of the fMet-Leu nascent chain connected to the A-tRNA (based on POST-state PDB ID: 1VY5)^35^. **e**, Steric clash of the fMet-Leu nascent chain in the P-site after translocation (based on PDB ID: 7RQE).

For the drosocin–ribosome complex containing A-site and P-site tRNAs, comparison of the cryo-EM density (Fig. 3a) with pre-attack and post-attack states^35^ (Fig. 3b,c) indicates that the P-site tRNA is deacylated, whereas the A-site tRNA carries a nascent chain (Fig. 3a). Thus, drosocin is bound to an elongating ribosome state that is post-peptide bond formation but pre-translocation. Inspection of the cryo-EM density for the anticodon–codon interactions suggested the presence of initiator tRNA^fMet^ and tRNA^Leu^ decoding the AUG and UUC codons in the first and second positions of the mRNA, respectively (Supplementary Fig. 3a,b). In this case, the nascent chain should comprise the dipeptide fMet-Leu, which is consistent with the limited space due to the presence of drosocin blocking the PTC and exit tunnel. However, because the density for the nascent chain was poorly resolved and, thus, could not be modeled de novo, only a tentative model for fMet-Leu could be generated to illustrate that the position is different than fMet-Phe in the post-peptide bond formation state reported previously (Fig. 3c,d)^35^. In the latter, we would predict steric clashes between the fMet moiety and the N-terminal Val19 of drosocin (Fig. 3c), which appears to have forced the fMet moiety to shift toward Arg18 (Fig. 3d), providing a likely explanation as to why both regions are poorly ordered in this complex (Fig. 3a). For the elongating complex to exist, drosocin allows initiation (despite predicted clashes between the fMet and Val19, as seen in Fig. 3b), aminoacyl-tRNA binding to the A-site and subsequent peptide bond formation, but it interferes with the first translocation step. To mimic the translocated state, we modeled fMet-Leu-tRNA bound in the P-site based on available P-site peptidyl-tRNAs^36^, which revealed even larger steric clashes with drosocin (Fig. 3e), providing a structural explanation for the observed translocation inhibition. We note that, although apidaecin strongly interferes with termination, moderate effects on initiation have also been reported in vivo and in vitro^37^. Given the similarity in the binding position of the C-terminus of Api137 and drosocin on the ribosome (Extended Data Fig. 2b), it seems likely that apidaecin may also interfere with the first translocation step, as seen here for drosocin, rather than acting like a type I PrAMP to prevent accommodation of the aminoacyl-tRNA at the A-site of the PTC, but this remains to be determined.

### Interaction of drosocin within the RF1-bound complex

**Extended Data Fig. 3:**
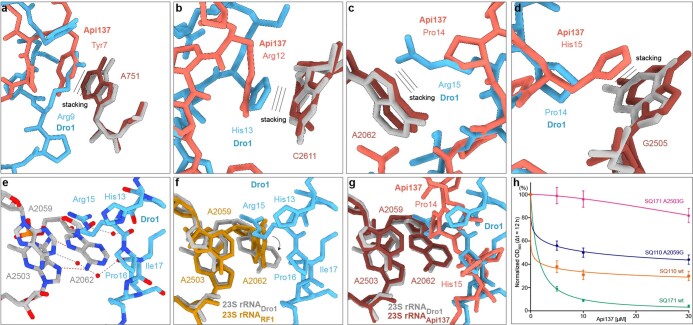
Comparison of Dro1 and Api137 interactions with the exit tunnel. **a-d**, Stacking interactions (indicated as three lines) of sidechains of Api137 (light red, PDB ID 5O2R)^10^ with 23S rRNA nucleotides (dark red) compared to Dro1 (light blue) stacking interactions with 23S rRNA nucleotides (grey). **e**, Water-mediated interactions of Dro1 (light blue) with surrounding 23S rRNA nucleotides (grey). (**f-g**) Comparison of Dro1 from (**e**) with (**f**) a canonical termination complex (PDB ID 4V63)^40^ and (**g**) Api137 (light red) and corresponding 23S rRNA nucleotides (dark red, PDB ID 5O2R)^10^. **h**, *in vivo* inhibitory activity of 5 μM, 10 μM and 30 μM Api137 on the growth of *E. coli* SQ110 wt (orange), *E. coli* SQ110 A2059G (blue), *E. coli* SQ171 wt (green) and *E. coli* SQ171 A2503G (pink) in LB medium. For each concentration, inhibition values are the OD_600_ at t = 12 h of the treated culture normalized to the untreated one, considered as 100 %. The plotted points represent the mean for three independent biological replicas, the error bars represent the standard deviation and the technical measurement error of the plate reader. The curves were calculated and plotted by non-linear regression. Source data

In the RF1-bound complex, Dro1 is very well resolved, enabling a molecular description of the interactions of the drosocin peptide with components of the ribosomal tunnel as well as RF1 (Supplementary Fig. 4a–g). Overall, there is excellent agreement between the interactions observed here for Dro1 and the extensive mutagenesis performed on Dro in ref. ^38^. In total, there are three stacking interactions observed between sidechains of Dro1 and the 23S rRNA—namely, between Arg9 and A751, His13 and C2611 as well as Arg15 and A2062. Mutation of Arg9 or Arg15 to lysine reduces antimicrobial activity of the Dro peptides by four-fold and eight-fold, respectively^39^, suggesting that these interactions contribute to drosocin binding. Api137 also stacks with A751 and C2611 (refs. ^10,11^); however, the sidechains and modes of interaction are completely distinct (Extended Data Fig. 3a–d). Compared to the canonical RF1-bound termination complexes^40,41^, we observed a rotated conformation of A2062, which was also observed in the Api137-bound ribosome structures^10,11^ (Extended Data Fig. 3e–g). The rotated conformation of A2062 forms interactions with A2503, which is adjacent to A2059, both of which were shown to confer resistance to Api137 when mutated^10^. Because Arg15 of Dro1 stacks upon A2062 (Supplementary Fig. 4g and Extended Data Fig. 3h) and is in close proximity to A2503 and A2059, we assessed whether A2503G and A2059G mutations confer resistance to Dro1. Indeed, we observed that, compared to the wild-type strain, both strains bearing the A2503G and A2059G mutations were more resistant to Api137 (Extended Data Fig. 3h), as previously reported^10^, but also to Dro1 (Supplementary Fig. 4h). We think that these findings provide strong evidence that the ribosome (and, therefore, translation) is a (if not ‘the’) physiological target for Dro1 within the bacterial cell. This is also supported by the identification of mutations in ribosomal protein uL16 that confer resistance to Api137 also confer resistance to Dro (see ref. ^38^).

### C-terminal interactions are critical for drosocin activity

**Fig. 4:**
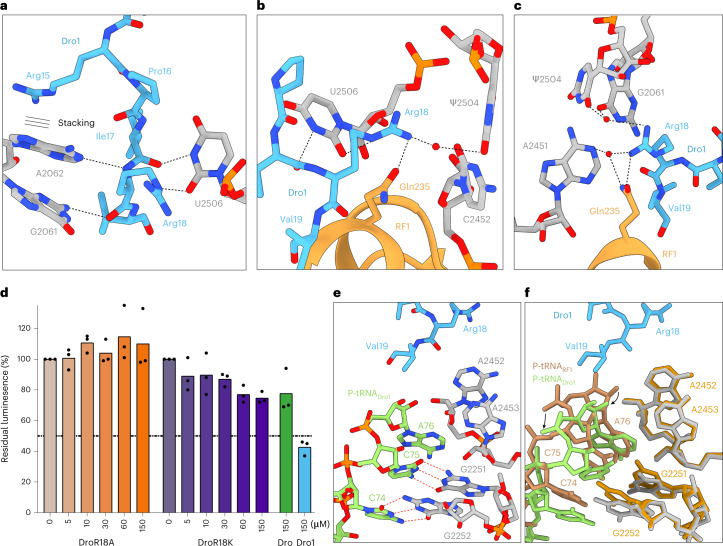
Interactions of drosocin with RF1 and P-tRNA. **a**–**c**, Interactions of Dro1 (light blue) with 23S rRNA nucleotides (gray) and RF1 (orange). **a**, Stacking interaction of Arg15 (indicated by three lines) and hydrogen bond interactions of G2061, A2062 and U2506 with Ile17 and Arg18 of Dro1. **b**,**c**, Two views of the water-mediated and direct hydrogen bond interactions of Arg18 of Dro1 with Gln235 of RF1 and 23S rRNA nucleotides Ψ2504, U2506 and C2452 (**b**) and G2061, A2451 and Ψ2504 (**c**). **d**, Inhibitory activity of increasing concentrations of Dro R18A (orange), Dro R18K (purple) and 150 μM Dro (green) or Dro1 (blue), on in vitro translation using Fluc as a reporter. The luminescence in the absence of compounds was normalized to 100%; experiments were performed in triplicate, individually plotted as dots, and the bars represent the mean. **e**, Deacylated P-tRNA (lime) in the presence of Dro1 (light blue). **f**, Superimposition of **e** with a P-tRNA from a canonical termination complex (brown; PDB ID: 4V63)^40^. Dro1 displaces the CCA-end of the deacylated tRNA while keeping the base-pairing interactions of C74 and C75 with G2252 and G2251 (gray), respectively, which slightly tilts the nucleotides compared to the canonical position (yellow). Source data

**Extended Data Fig. 4:**
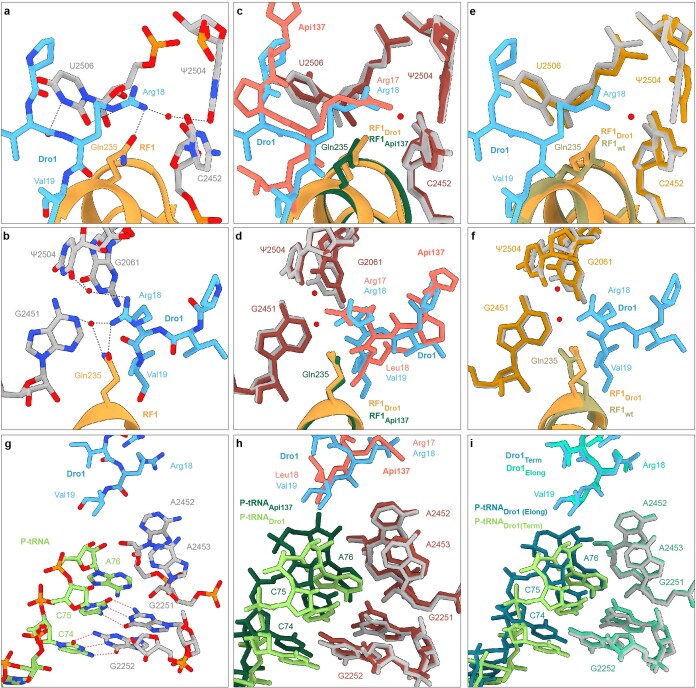
Interaction of RF1 and P-tRNA with Dro1. **a-b**, Water-mediated and direct hydrogen bond interactions of Arg18 of Dro1 (light blue) with surrounding 23S rRNA nucleotides (grey) and Gln235 of RF1 (orange) from two views. **c-f**, Comparison of (**a-b**) with (**c-d**) Api137 (light red, PDB ID 5O2R)^10^ with RF1 (dark green) and 23S rRNA (dark red) and with (**e-f**) a canonical termination complex RF1 (olive, PDB ID 4V63)^40^ and surrounding 23S rRNA nucleotides (yellow). **g**, CCA-end of a deacylated tRNA (lime) in the P-site in presence of Dro1 is shifted while still establishing base-pairing interactions with G2251 and G2252. **h-i**, Comparison of (**g**) with superimposed deacylated tRNA in the P-site (dark green) and (**h**) the presence of Api137 (light red, PDB ID 5O2R)^10^ or (**i**) the drosocin-bound elongation complex with deacylated tRNA in the P-site (dark teal) and Val19 of Dro1 poorly ordered (teal).

The C-terminus of Dro1 is stabilized by three backbone interactions between residues Ile17–Arg18 and 23S rRNA nucleotides U2506, G2061 and A2062 (Fig. 4a). Additionally, the sidechain of Arg18 inserts into a pocket where it can form direct hydrogen bonds with the nucleobases of C2452 and U2506 (Fig. 4b) as well as via water-mediated interactions with Ψ2504, G2061 and A2451 (Fig. 4b,c). Notably, Arg18 comes within 2.9 Å of Gln235 of the conserved GGQ motif of RF1, and a further water-mediated interaction with Gln235 is also possible (Fig. 4b,c), suggesting that Arg18 plays an important role in stabilizing RF1 on the ribosome. This interaction is reminiscent of that observed previously between Arg17 of Api137 and Gln235 of RF1 (refs. ^10,11^) (Extended Data Fig. 4a–f), the importance of which was shown by Arg17Ala mutations that decrease both the ribosome affinity and inhibitory activity of the peptide^2^. Although deletion of the last two residues (Arg18–Val19) of drosocin completely abolished in vitro biological activity^16^, single substitutions of Arg18 have, to our knowledge, not been undertaken. Therefore, we synthesized an unmodified drosocin peptide bearing the Arg18Ala mutation (Fig. 1a) and tested its activity using in vitro translation assays, demonstrating a complete loss of activity for the Dro-R18A peptide (Fig. 4d). By contrast, Dro bearing an Arg18Lys mutation (Dro-R18K; Fig. 1a) displayed similar activity to Dro (Fig. 4d). Unlike Arg18, the very C-terminal Val19 of Dro1 is poorly ordered, but, at lower thresholds, density is observed to encroach on the P-site of the PTC (Fig. 4e,f). As a consequence, the CCA-end of the P-site tRNA, which is also poorly resolved, is clearly shifted by 2–3 Å from its canonical position observed in RF1 termination complexes^40,41^ (Fig. 4f). The shift is predominantly of the backbone of the CCA-end enabling the nucleobases of C74 and C75 to maintain Watson–Crick base pairs with P-loop nucleotides G2252 and G2251, respectively (Fig. 4e,f). This is distinct from Api137, where the C-terminus was observed to directly interact with the A76 of the P-site tRNA and stabilize the P-site tRNA in its canonical position (Extended Data Fig. 4g–h). By comparison, we did not observe a shifted P-site tRNA in the Dro1-bound elongating state (Extended Data Fig. 4i). Otherwise, the binding position and interactions of RF1 in the Dro1–RF1–ribosome complex are identical to those observed previously for RF1 decoding of stop codons during canonical termination^40,41^ (Supplementary Fig. 5a–c). However, with the higher resolution, we also observed multiple water-mediated interactions between RF1 and the UAA stop codon (Supplementary Fig. 5d–g), which were not reported in the previous lower-resolution termination complexes^40,41^.

### Interaction of drosocin O-glycosylation with the ribosome

**Fig. 5:**
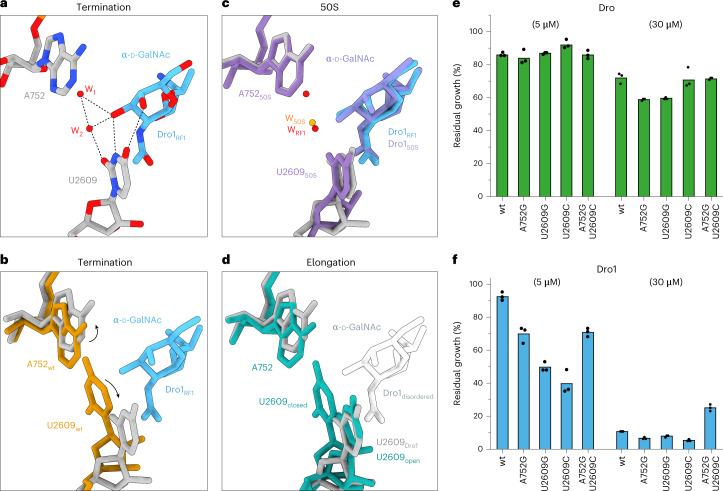
Interaction of O-glycosylation of Dro1 with U2609 of the 23S rRNA. **a**, Molecular interactions between the α-d-GalNAc modification on Thr11 of Dro1 (light blue) and the 23S rRNA nucleotide U2609 (gray) of the Dro1-bound termination complex. Two coordinated water molecules (red) stabilize the interactions of α-d-GalNAc of Dro1 with U2609. **b**–**d**, Superimposition of Dro1_RF1_ (light blue), waters (red) and 23S rRNA (gray) from **a** with 23S rRNA (yellow) from canonical RF1-bound termination complex (PDB ID: 4V63)^40^ (**b**), 23S rRNA (purple) from the Dro1-bound 50S complex (**c**) and 23S rRNA (turquoise) from the Dro1-bound elongation complex with two alternative conformations (open and closed) of U2609 shown (**d**). α-d-GalNAc modification of Dro1 was poorly ordered in the elongation complex; therefore, the white silhouette indicates the position from Dro1_RF1_ that is incompatible with the closed conformation of U2609. **e**,**f**, In vivo inhibitory activity of 5 μM and 30 μM of Dro (green) (**e**) and Dro1 (blue) (**f**) on the growth of *E. coli* SQ171 wild-type, *E. coli* SQ171 A752G, *E. coli* SQ171 U2609G, *E. coli* SQ171 U2609C and *E. coli* SQ171 A752G/U2609C. Histograms represent the averages from three biological replicates, individually plotted as dots. wt, wild-type. Source data

**Extended Data Fig. 5:**
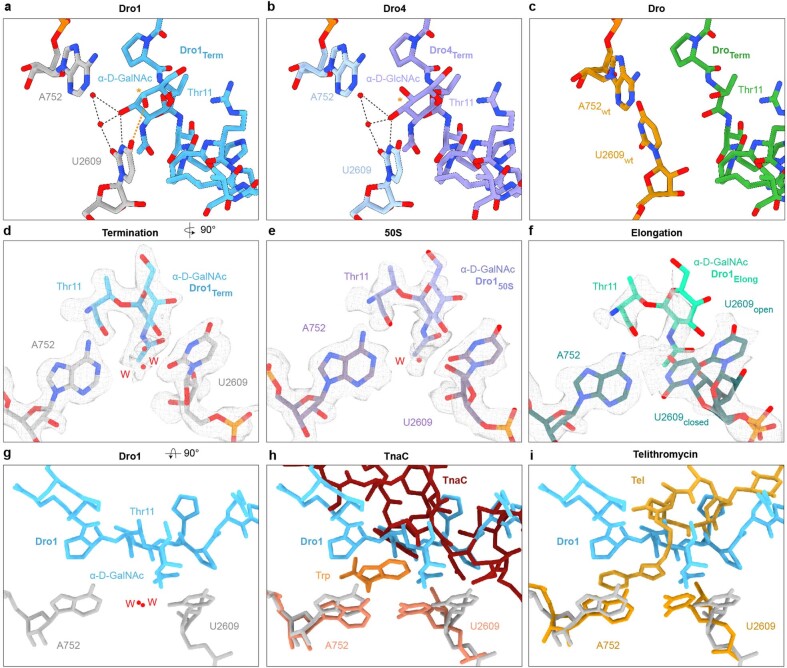
Sugar modification of drosocin. **a-c**, Comparison of (**a**) the α-D-GalNAc modification of Dro1 (light blue) in the termination complex with (**b**) an *in silico* model for the α-D-GlcNAc modification of Dro4 (**b**, light purple) indicating potential loss of a weak hydrogen bond (orange) of C4 hydroxy group of the sugar with C4 hydroxy group of U2609 (grey) as well as (**c**) comparison with an *in silico* model for the unmodified Dro that lacks the Thr-11 glycosylation (green). **d-f**, Isolated density (mesh) for Thr-11 with sugar modification and 23S rRNA nucleotides A752 and U2609 in an open conformation for the (**d**) termination (grey) and (**e**) 50S complexes and (**f**) both open and closed conformations for the elongation complex (dark green). **g-i**, Dro1 (light blue) with 23S rRNA nucleotides A752 and U2609 (grey) and waters (red) in the termination complex superimposed with (**h**) TnaC (red) with 23S rRNA nucleotides (salmon) and Tryptophan (orange) from the TnaC-stalled *E. coli* ribosome complex (PDB ID 7O19)^44^, and with (**i**) telithromycin (Tel, yellow, PDB ID 4V7S)^42^ with 23S rRNA nucleotides (orange).

For the Dro1–RF1–70S and Dro1–50S complexes, the α-d-GalNAc modification linked to Thr11 establishes multiple interactions with U2609 of the 23S rRNA (Fig. 5a). In particular, the C3 hydroxyl comes within 2.6 Å and 2.7 Å of the N3 and O2, respectively, of the base of U2609 (Fig. 5a). Additionally, a hydrogen bond is also possible (3.5 Å) from the C4 hydroxyl to the O4 of U2609 (Fig. 5a). We note that α-d-GlcNAc present in Dro4 would maintain the former interactions and lose only the latter weaker interaction with O4 of U2609 (Extended Data Fig. 5a,b), consistent with the similar activity of Dro4 compared to Dro1 (Fig. 1c,d). By contrast, modifications of β-d-linkage, as in Dro3 and Dro5–Dro8, would be incompatible with the interactions observed with α-d-GlcNAc, providing an explanation for why they exhibit lower activity compared to Dro1 and Dro4 (Fig. 1c,d). Comparison with other *E. coli* 70S ribosome structures, including RF1 termination complexes^40,41^, reveals that U2609 is usually base paired with A752 (Fig. 5b and Extended Data Fig. 5c), whereas, in the Dro1–RF1–70S and Dro1–50S complexes, α-d-GalNAc occupies the position of U2609, causing the base to shift away from A752 by up to 6 Å (Fig. 5b,c and Extended Data Fig. 5d,e). Moreover, we observed two water molecules located between U2609 and A752 that may also contribute to stabilizing the shifted conformation by establishing indirect interactions between U2609 and α-d-GalNAc (Fig. 5a,c). Interestingly, in the cryo-EM map of Dro1 bound to the elongating ribosome, we observed both the base-paired and shifted conformation of U2609 (Fig. 5d and Extended Data Fig. 5f). As mentioned, the density for α-d-GalNAc is less well resolved in this complex (Fig. 2h and Extended Data Fig. 5f), suggesting that it is highly flexible, presumably because it cannot adopt the preferred position interacting with the shifted conformation of U2609.

Collectively, these findings suggest that the propensity of U2609 and A752 to base pair could influence the ability of Dro1 to bind stably to the ribosome and inhibit translation. To test this, we monitored the antimicrobial activity of Dro1 on strains bearing A752G, U2609G or U2609C mutations, which should perturb Watson–Crick base pairing. In addition, we also used a strain with a U2609C–A752G double mutation, which would be predicted to restore Watson–Crick base pairing, and with three hydrogen bonds could possibly make breaking the base pair harder than with the canonical two hydrogen bonds for the A–U base pair. As a control, we also tested Dro that lacks the α-d-GalNAc modification, which we predict (assuming that Dro binds analogously to Dro1) should not interact with U2609 and, therefore, not be influenced by the conformation of the U2609–A752 base pair. As seen in Fig. 5e, we observed that there was no significant difference in growth inhibition by 5 μM Dro and only a modest effect at 30 μM Dro, when comparing the wild-type strain and strains bearing single or double mutations. By contrast, we observed that the growth of the strains bearing the single point mutations was more susceptible to Dro1 than the wild-type strain, especially for the U2609C mutation, although this effect became less evident at higher (30 μM) drug concentrations (Fig. 5f). Although the U2609C–A752G double mutation was also slightly more susceptible than the wild-type to Dro1 at 5 μM, it was still less susceptible than most single point mutations and appeared to be 2.5-fold more tolerant to Dro1 than the wild-type strain at 30 μM (Fig. 5f). Collectively, these findings support a role for the U2609–A752 base pair in modulating the ribosome binding and inhibition activity of glycosylated drosocin.

## Discussion

**Fig. 6:**
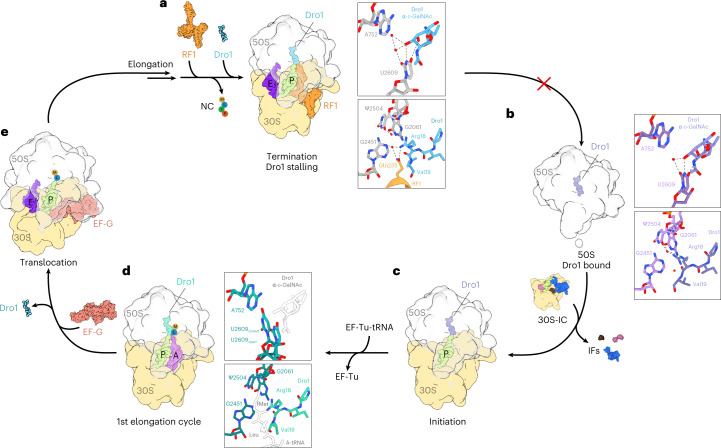
Model for the mechanism of action of Dro1 inhibition during translation. **a**, Appearance of a stop codon in the A-site is recognized by RF1 (or RF2, orange), which catalyzes release of the nascent chain (NC) from the P-site tRNA (lime). After NC release, Dro1 (light blue) binds within the exit tunnel, separating the A752–U2609 base pair (gray) with Dro1 α-d-GalNAc modification and becomes stabilized via water-mediated and direct interactions between Arg18 of Dro1 and the Gln235 of the conserved GGQ motif of RF1 and surrounding 23S rRNA nucelotides. This interaction stabilizes RF1 on the post-release complex, preventing its dissociation and, thereby, blocking subsequent ribosome recycling steps and re-initiation. **b**, Dro1 (purple) binds to free 50S subunits (gray), separating the A752–U2609 base pair (light purple) with Dro1 α-d-GalNAc modification but is not fully stabilized via water-mediated and direct interactions between Arg18 of Dro1 and surrounding 23S rRNA nucleotides. **c**, Translation initiation complexes can form in the presence of Dro1 (purple), despite slight overlap between Dro1 and the fMet moiety of the P-tRNA (lime), suggesting that fMet might displace the C-terminal part of Dro1. **d**, After peptide bond formation, the presence of Dro1 (teal) appears to interfere with translocation of the dipeptidyl-tRNA in the A-site (purple) into the P-site (lime). The α-d-GalNAc modification (white) is disordered, and both the open and closed conformation of the U2609 base (dark teal) is observed. The dipeptidyl moiety (white) on the A-tRNA interferes with the stabilization of Dro1 in the PTC. **e**, For translocation to occur, and subsequent steps of elongation to occur, Dro1 must dissociate from the ribosome, followed by elongation until translation termination is reached.

Our biochemical and structural analysis allows us to propose a model for the mechanism of action of drosocin, highlighting the role of the O-glycosylation (Fig. 6). Analogous to Api137 (refs. ^10,11^), we reveal that drosocin interferes with the translation termination by trapping RF1 on the ribosome subsequent to the release of the nascent polypeptide chain (Fig. 6a). Like Api137 (refs. ^10,11^), the C-terminal Arg18 of Dro directly interacts with Gln235 of the conserved GGQ motif of RF1 (Fig. 6a). Arg18 of Dro is critical because mutation to alanine abolishes all inhibitory activity of the peptide (Fig. 4d), collectively providing a structural basis for how RF1 dissociation is impeded by drosocin. Unlike Api137, drosocin is O-glycosylated on Thr11, and we observed that the α-d-GalNAc modification contributes to the ribosome binding by establishing multiple hydrogen bond interactions with U2609 of the 23S rRNA (Fig. 6a). These interactions rationalize our (Fig. 1) and previous^17–24^ observations that the native modified forms of drosocin generally display enhanced antimicrobial activity compared to the unmodified peptide. Interestingly, we observed that drosocin causes a shift of U2609 that breaks the base pair that U2609 usually forms with A752 (Fig. 6a). Consistently, we could demonstrate that single and double mutations at these positions could influence the activity of the glycosylated, but not the unmodified, form of drosocin (Fig. 5e,f). To our knowledge, breaking of this base pair has not been observed in *E. coli* previously, although the base pair is important for interaction^42^ and bactericidal activity^43^ of the ketolide telithromycin and also interacts with the free tryptophan during TnaC-mediated translational stalling^44^ (Extended Data Fig. 5g–i). We note, however, that U2609 and A752 are unpaired in some bacterial ribosomes, such as *Mycobacterium tuberculosis*^45^, raising the question of whether these ribosomes are more susceptible to glycosylated forms of drosocin.

In addition to the termination complex, we observed drosocin bound to two other ribosomal particles—namely, a vacant 50S subunit and an elongating ribosome (Fig. 2b,c). This implies that, in the cell, drosocin could potentially interact with the 50S subunit after termination and ribosome recycling, when the 70S ribosomes are split into their component subunits (Fig. 6b). This is not surprising given that most of the interactions formed by drosocin are identical between the vacant and terminating ribosome. Indeed, we observed that, on the vacant 50S ribosome, the α-d-GalNAc has also inserted in between the U2609–A752 base pair, causing a shift in U2609 as observed in the termination state (Fig. 6b). By contrast, the C-terminus of drosocin on the vacant 50S subunit appears flexible and less well resolved, presumably because the interaction with Gln235 of RF1 is absent (Fig. 6b). Similarly, binding of Api137 has previously been shown to be stabilized on 70S ribosomes by the presence of RF1 when compared to vacant ones^10^. Because we observed no initiation states within our structural ensembles, we presume that the fMet-tRNA can bind at the P-site of the PTC unimpeded by the presence of drosocin (Fig. 6c), possibly by competing with the C-terminus for its binding site at the PTC. Conversely, we observed a major population of drosocin-bound ribosomes that was in an elongation state—namely, a post-peptide bond formation pre-translocation state with deacylated-tRNA^fMet^ in the P-site and a fMet-Leu-tRNA^Leu^ in the A-site (Fig. 6d). This suggests that drosocin can interfere with the first translocation event involving the movement of the fMet-Leu-tRNA^Leu^ into the P-site. We think that this arrest is likely to be temporary because, in our toeprinting experiments, we observed that ribosomes can eventually translate the entire open reading frame (ORF) and become trapped at the termination codon (Fig. 1f–h). In the elongation state, drosocin is particularly flexible and poorly resolved, which is exemplified by the poor density for the α-d-GalNAc and the presence of both closed (base paired) and open (unpaired) conformations of U2609 (Fig. 6d and Extended Data Fig. 5a–f). We favor a model whereby drosocin and the fMet-Leu-tRNA^Leu^ jostle for position at the P-site of the PTC and that occupation by fMet-Leu-tRNA^Leu^ triggers translocation and subsequent rounds of elongation that ultimately cause dissociation of drosocin from the ribosome (Fig. 6e). Once the nascent polypeptide chain becomes extended within the ribosomal tunnel, drosocin cannot rebind until the termination codon is reached and the nascent chain is released by RF1 (or RF2) (Fig. 6a).

It is remarkable that, although both drosocin and apidaecin inhibit translation by trapping RFs on the ribosome in an analogous manner, the binding mode and molecular details of the interactions of these peptides with components of the ribosomal tunnel are completely distinct. This is accentuated by the presence of O-glycosylation that plays a critical role for drosocin but is lacking for apidaecin. Curiously, other AMPs are glycosylated, such as pyrrhocoricin^13^, which bears an identical modification to drosocin at exactly the same position—namely, GalNAc on Thr11—and minor forms with an additional galactose on the GalNAc have been also detected^27^. Although structures of the unmodified pyrrhocoricin on the ribosome reveal a reversed orientation compared to drosocin^6,7^, superimposition reveals that Thr11 of pyrrhocoricin and drosocin are in close proximity, raising the possibility that the glycosylation of pyrrhocoricin may establish analogous interactions with the ribosome, as observed here for drosocin. Finally, we show that drosocin traps RF1 decoding the UAA stop codon on the ribosome in an analogous manner to that observed during canonical translation. However, the higher resolution observed here enables us to observe many water-mediated interactions that were not possible to observe previously. Thus, our study also provides structural insight into the fundamental mechanism of stop codon recognition during canonical translation termination.

## Methods

### Drosocin peptides

Api137, Dro, Dro-3P, Dro-R18A and Dro-R18K were synthesized by NovoPro (https://www.novoprolabs.com). The glycosylated Dro1–Dro8 peptides were synthesized as described^23–25,46^.

### Bacterial strains

Strains *E. coli* Keio wild-type and *E. coli* Keio Δ*sbmA* used from the Keio knockout collection (Horizon, https://horizondiscovery.com). Wild-type *E. coli* SQ110 (Δ*rrn*GADBHC(ptRNA67))^47^ and SQ171 (Δ*rrn*GADEHBC(pKK3535, ptRNA67))^47^ strains and related mutants *E. coli* SQ110 A2059G and *E. coli* SQ171 A2503G were obtained from the previous Api137 study^10^. *E. coli* strains SQ171 bearing A752G, U2609C and A752G:U2609C mutations^43^ and *E. coli* SQ171 U2609G^48^ were generated previously.

### Antibiotic susceptibility assays

The susceptibility of *E. coli* strains to compounds was evaluated by monitoring the bacterial growth in presence of increasing concentrations of the compound of interest. In brief, bacteria were inoculated in a total volume of 100 μl of medium contained in a well of a 96-well microplate (round bottom, with cap, sterile; Sarstedt). The medium used was either LB, as rich medium, or ATCC medium (778 Davis and Mingioli glucose minimal medium), as minimal medium. Before inoculation, bacteria were grown up to exponential phase and then inoculated into the culture mix, containing selective antibiotic if necessary, with an initial optical density at 600 nm (OD_600_) of 0.05. Values measured from wells containing just the medium were used as a blank. The growth in each well was monitored by measuring the OD_600_ every 10 minutes for a total of 20 hours at 37 °C with shaking using a plate reader (Tecan Infinite 200 PRO). The inhibition resulting from a compound’s concentration was evaluated by normalizing the OD_600_ at *t* = 12 hours (corresponding to the end of the log phase) from the treated culture to the untreated one. For each compound, the concentrations tested were 5 μM, 10 μM and 30 μM. Each single titration assay was done in triplicate with individually prepared culture mixes. For each concentration, the standard deviation was calculated, taking into account each single replica and its specific technical error from the plate reader.

### Data analysis

Data from the in vivo assay were normalized and statistically analysed by GraphPad Prism version 9.4.0.

### In vitro translation assays

The in vitro translation assay was carried out as described previously^4,8^ using the *E. coli* PURExpress system (New England Biolabs (NEB), E6800S). Then, 1 µl of antibiotic solution was added to 5 µl of PURExpress reaction mix. Each reaction contained 10 ng μl^−1^ of mRNA encoding the Fluc, which was in vitro transcribed from a pIVEX-2.3MCS vector containing the Fluc gene using T7 polymerase (Thermo Fisher Scientific). The reaction mix was incubated for 30 minutes at 32 °C while shaking (600 r.p.m.). Reactions were stopped with 5 µl of kanamycin (50 mg ml^−1^) and transferred into a 96-well microplate (Greiner Lumitrac, non-binding, white, chimney). Next, 40 µl of luciferase assay substrate solution (Promega, E1501) was added, and luminescence was measured using a plate reader (Tecan Infinite 200 PRO). Nuclease-free water was added instead of antibiotic as control. Absolute luminescence values were normalized using reactions without antibiotic. All assays were done as triplicates with individually prepared reaction mix.

### Toeprinting assays

Toeprinting reactions were performed as described previously^4^. In brief, reactions were performed with 6 μl of PURExpress ΔRF123 in vitro protein synthesis system (NEB) in the presence of 1× RF3 and either 1× or 10× of RF1 or RF2 (relative to the manufacturer’s recommendation). The reactions were carried out on an MLIF-UAA-toeprint template (5′-TAATACGACTCACTATAGGGAGACTTAAGTATAAGGAGGAAAAAAT**ATG**ATATTCTTG**TAA**ATGCGTAATGTAGATAAAACATCTACTATTTAAGTGATAGAATTCTATCGTTAATAAGCAAAATTCATTATAACC-3′, ORF start codon and stop codon are underlined bold), containing T7 promoter, a ribosome binding site, an MLIF-coding ORF and the NV1* primer binding site. The template is a version of the ErmBL template with a truncated ORF and addition of a isoleucine coding codon at the third position in the ORF. The template was generated by polymerase chain reaction of two overlapping 77-nt- and 78-nt-long primers. The reactions contained 30 ng of the MLIF-UAA-toeprint DNA template. The reactions were supplemented with Api137, thiostrepton or one of the drosocin derivates as specified. The transcription–translation reactions were incubated for 15 minutes at 37 °C. The reverse transcription on the MLIF-short-UAA toeprint template was carried out using AMV RT and primer NV*1-Alexa 647 (5′-GGTTATAATGAATTTTGCTTATTAAC-3′). The transcription–translation reactions were incubated with AMV RT and NV*1-Alexa 647 for 20 minutes at 37 °C. mRNA degradation was carried out by the addition of 1 µl of 5 M NaOH. The reactions were neutralized with 0.7 µl of 25% HCl, and nucleotide removal was performed with the QIAquick Nucleotide Removal Kit (Qiagen). The samples were dried under vacuum for 2 hours at 60 °C for subsequent gel electrophoresis. The 6% acrylamide gels were scanned on a Typhoon scanner (GE Healthcare).

### Preparation of complexes for structural analysis

Drosocin–ribosome complexes were generated by in vitro transcription–translation reactions in PURExpress ΔRF123 in vitro protein synthesis system (NEB) with the same reaction mix as described earlier in the toeprinting assays. Complex formation reactions were carried out on an MLIF-UAA toeprint DNA template in a 48-µl reaction with 1× RF3 and 10× RF1 (amounts relative to the manufacturer’s recommendation) in the presence of 30 µM Dro1. The reaction was incubated for 15 minutes at 37 °C. The reaction volume was then split: 42 µl was used for complex generation, and 6 µl was used for toeprinting analysis. Ribosome complexes were isolated by centrifugation in 900 µl of sucrose gradient buffer (containing 40% sucrose, 50 mM HEPES-KOH pH 7.4, 100 mM KOAc, 25 mM Mg(OAc)_2_ and 6 mM 2-mercaptoethanol) for 3 hours at 4 °C and 80,000*g* in an Optima Max-XP Tabletop Ultracentrifuge with a TLA 120.2 rotor. The pelleted complex was resuspended in Hico buffer (50 mM HEPES-KOH pH 7.4, 100 mM KOAc and 25 mM Mg(OAc)_2_ supplemented with RF1, RF3 and Dro1 at the same concentrations used in the in vitro translation reaction) and then incubated for 15 minutes at 37 °C.

### Preparation of cryo-EM grids and data collection

Grids (Quantifoil R3/3 Cu300 with 3-nm holey carbon) were glow discharged, and 4 µl of sample (8 OD_260_ per milliliter) was applied using a Vitrobot Mark IV (FEI) and snap-frozen in ethane/propane. Frozen cryo-EM grids were imaged on a TFS 300 kV Titan Krios at the Dubochet Center for Imaging EPFL (Lausanne, Switzerland). Images were collected on a Falcon IV direct detection camera in counting mode using the EPU and AFIS data collection scheme with a magnification of ×96,000 and a total dose of 40 electrons per square angstrom (e^−^/Å^2^) for each exposure and defocus ranging from −0.4 µm to −0.9 µm. In total, 8,861 movies were produced in electron event representation format.

### Single-particle reconstruction of drosocin–ribosome complexes

RELION version 4.0 (ref. ^49^) was used for processing, unless otherwise specified. For motion correction, RELION’s implementation of MotionCor2 with 4 × 4 patches, and, for initial contrast transfer function (CTF) estimation, CTFFIND version 4.1.14 (ref. ^50^), were employed. From 8,861 micrographs, 715,455 particles were picked using crYOLO with a general model^51^. In total, 529,600 ribosome-like particles were selected after two-dimensional (2D) classification and extracted at 3× decimated pixel size (2.4 Å per pixel) (Supplementary Fig. 2). An initial three-dimensional (3D) refinement was done using an *E. coli* 70S reference map (EMD-12573) and followed by initial 3D classification without angular sampling with six classes. Two classes containing 70S ribosomes were combined (356,671 particles) and subsorted. A class containing 50S subunits (159,749 particles) was further processed. We observed no classes containing RF3, despite the presence of RF3 in the translation reactions. However, unlike our previous study^11^, we did not use non-hydrolysable GTP analogs. The subsorting was done using particle subtraction with a circular mask around the A-site with four classes. One class containing density that could be assigned RF1 (137,449 particles), and one class with A-tRNA density (84,697 particles), were further processed. All resulting classes were 3D refined and CTF refined (4^th^ order aberrations, beam tilt, anisotropic magnification and per-particle defocus value estimation). The termination complex was additionally subjected to Bayesian polishing and another round of CTF refinement. For the termination, elongation and 50S complexes, final resolutions (gold-standard FSC_0.143_) of masked reconstructions of 2.3 Å, 2.8 Å and 2.0 Å were achieved, respectively (Extended Data Fig. 1a–c). To estimate local resolution values, Bsoft^52^ was used on the half-maps of the final reconstructions (blocres -sampling 0.8 -maxres -boc 20 -cutoff 0.143 -verbose 1 -origin 0,0,0 -Mask half_map1 half_map 2) (Extended Data Fig. 1d–i).

### Molecular modeling of the drosocin–ribosome complexes

The molecular models of the 30S and 50S ribosomal subunits were based on the *E. coli* 70S ribosome (Protein Data Bank (PDB) ID: 7K00)^53^. Drosocin was modeled de novo, and the 2-acetamido-2-deoxy-α-d-galactopyranose was taken from the Ligand Expo database A2G (PDB ID: 1D0H) and linked through REFMAC 5 (ref. ^54^). Restraint files for modified residues were created using aceDRG^55^. The termination complex was assembled with an RF1 AlphaFold model (AF-P0A7I0-F1) and a crystal structure of a deacylated phenylalanine tRNA (PDB ID: 6Y3G) in the P-site. The elongation complex was assembled with an initiator fMet-tRNA (PDB ID: 1VY4)^35^ in the P-site and a Leu-tRNA (PDB ID: 7NSQ) in the A-site. Starting models were rigid body fitted using ChimeraX^56^ and modeled using Coot 0.9.8.3 (ref. ^57^) from the CCP4 software suite version 8.0 (ref. ^58^). The sequence for the tRNAs was adjusted based on the appropriate anticodons corresponding to the mRNA. Final refinements were done in REFMAC 5 (ref. ^54^) using Servalcat^59^. The molecular models were validated using Phenix comprehensive cryo-EM validation in Phenix 1.20–4487 (ref. ^60^).

### Figures

UCSF ChimeraX 1.3 was used to isolate density and visualize density images and structural superpositions. Models were aligned using PyMol version 2.4 (Schrödinger). Figures were assembled with Adobe Illustrator and Inkscape (latest development release, regularly updated).

### Reporting summary

Further information on research design is available in the Nature Portfolio Reporting Summary linked to this article.

## Online content

Any methods, additional references, Nature Portfolio reporting summaries, source data, extended data, supplementary information, acknowledgements, peer review information; details of author contributions and competing interests; and statements of data and code availability are available at 10.1038/s41589-023-01293-7.

## Supplementary information

Supplementary Information Supplementary Figs. 1–5, Table 1 and References.

Reporting Summary

Supplementary Data Statistics for Supplementary Fig. 4h.

## Data Availability

Micrographs have been deposited as uncorrected frames in the Electron Microscopy Public Image Archive with accession code EMPIAR-11388. Cryo-EM maps have been deposited in the Electron Microscopy Data Bank with accession codes EMD-15488 (drosocin termination complex), EMD-15523 (drosocin elongation complex) and EMD-15533 (drosocin 50S complex). Molecular models have been deposited in the Protein Data Bank with accession codes 8AKN (drosocin termination complex), 8AM9 (drosocin elongation complex) and 8ANA (drosocin 50S complex). Source data are provided with this paper.

## Source data

Source Data Fig. 1 Statistical data for Fig. 1c–e,f–h.
Source Data Fig. 4 Statistical data for Fig. 4d.
Source Data Fig. 5 Statistical data for Fig. 5e,f.
Source Data Extended Data Fig. 3 Statistical data for Extended Data Fig. 3h.

## References

[1] Mardirossian M (2014). The host antimicrobial peptide Bac71-35 binds to bacterial ribosomal proteins and inhibits protein synthesis. Chem. Biol..

[2] Krizsan A (2014). Insect-derived proline-rich antimicrobial peptides kill bacteria by inhibiting bacterial protein translation at the 70S ribosome. Angew. Chem. Int. Ed. Engl..

[3] Graf M, Wilson DN (2019). Intracellular antimicrobial peptides targeting the protein synthesis machinery. Adv. Exp. Med. Biol..

[4] Seefeldt AC (2015). The proline-rich antimicrobial peptide Onc112 inhibits translation by blocking and destabilizing the initiation complex. Nat. Struct. Mol. Biol..

[5] Roy RN, Lomakin IB, Gagnon MG, Steitz TA (2015). The mechanism of inhibition of protein synthesis by the proline-rich peptide oncocin. Nat. Struct. Mol. Biol..

[6] Seefeldt AC (2016). Structure of the mammalian antimicrobial peptide Bac7(1–16) bound within the exit tunnel of a bacterial ribosome. Nucleic Acids Res..

[7] Gagnon MG (2016). Structures of proline-rich peptides bound to the ribosome reveal a common mechanism of protein synthesis inhibition. Nucleic Acids Res..

[8] Mardirossian M (2018). The dolphin proline-rich antimicrobial peptide Tur1A inhibits protein synthesis by targeting the bacterial ribosome. Cell Chem. Biol..

[9] Mardirossian M (2020). Peptide inhibitors of bacterial protein synthesis with broad spectrum and SbmA-independent bactericidal activity against clinical pathogens. J. Med. Chem..

[10] Florin T (2017). An antimicrobial peptide that inhibits translation by trapping release factors on the ribosome. Nat. Struct. Mol. Biol..

[11] Graf M (2018). Visualization of translation termination intermediates trapped by the apidaecin 137 peptide during RF3-mediated recycling of RF1. Nat. Commun..

[12] Bulet P (1993). A novel inducible antibacterial peptide of *Drosophila* carries an O-glycosylated substitution. J. Biol. Chem..

[13] Bulet P, Hetru C, Dimarcq JL, Hoffmann D (1999). Antimicrobial peptides in insects; structure and function. Dev. Comp. Immunol..

[14] Uttenweiler-Joseph S (1998). Differential display of peptides induced during the immune response of *Drosophila*: a matrix-assisted laser desorption ionization time-of-flight mass spectrometry study. Proc. Natl Acad. Sci. USA.

[15] Rabel D (2004). Primary structure and in vitro antibacterial properties of the *Drosophila melanogaster* attacin C pro-domain. J. Biol. Chem..

[16] Hoffmann R, Bulet P, Urge L, Otvos L (1999). Range of activity and metabolic stability of synthetic antibacterial glycopeptides from insects. Biochim. Biophys. Acta.

[17] Gobbo M (2002). Antimicrobial peptides: synthesis and antibacterial activity of linear and cyclic drosocin and apidaecin 1b analogues. J. Med. Chem..

[18] Marcaurelle LA, Rodriguez EC, Bertozzi C (1998). Synthesis of an oxime-linked neoglycopeptide with glycosylation-dependent activity similar to its native counterpart. Tetrahedron Lett..

[19] Rodriguez EC, Winans KA, King DS, Bertozzi CR (1997). A strategy for the chemoselective synthesis of O-linked glycopeptides with native sugar–peptide linkages. J. Am. Chem. Soc..

[20] Otvos L (2000). Interaction between heat shock proteins and antimicrobial peptides. Biochemistry.

[21] Ahn M (2011). Substitution of the GalNAc-α-*O*-Thr^11^ residue in drosocin with *O*-linked glyco-peptoid residue: effect on antibacterial activity and conformational change. Bioorg. Med. Chem. Lett..

[22] Ahn M (2011). Functional and structural characterization of drosocin and its derivatives linked O-GalNAc at Thr^11^ residue. Bull. Korean Chem. Soc..

[23] Talat S, Thiruvikraman M, Kumari S, Kaur KJ (2011). Glycosylated analogs of formaecin I and drosocin exhibit differential pattern of antibacterial activity. Glycoconj. J..

[24] Lele DS, Dwivedi R, Kumari S, Kaur KJ (2015). Effect of distal sugar and interglycosidic linkage of disaccharides on the activity of proline rich antimicrobial glycopeptides. J. Pept. Sci..

[25] Lele DS, Talat S, Kumari S, Srivastava N, Kaur KJ (2015). Understanding the importance of glycosylated threonine and stereospecific action of drosocin, a proline rich antimicrobial peptide. Eur. J. Med. Chem..

[26] Ludwig T, Krizsan A, Mohammed GK, Hoffmann R (2022). Antimicrobial activity and 70S ribosome binding of apidaecin-derived Api805 with increased bacterial uptake rate. Antibiotics (Basel).

[27] Cociancich S (1994). Novel inducible antibacterial peptides from a hemipteran insect, the sap-sucking bug *Pyrrhocoris apterus*. Biochem. J..

[28] Krizsan A, Prahl C, Goldbach T, Knappe D, Hoffmann R (2015). Short proline-rich antimicrobial peptides inhibit either the bacterial 70S ribosome or the assembly of its large 50S subunit. ChemBioChem.

[29] Berthold N, Hoffmann R (2014). Cellular uptake of apidaecin 1b and related analogs in Gram-negative bacteria reveals novel antibacterial mechanism for proline-rich antimicrobial peptides. Protein Pept. Lett..

[30] Benincasa M (2004). Antimicrobial activity of Bac7 fragments against drug-resistant clinical isolates. Peptides.

[31] Mattiuzzo M (2007). Role of the *Escherichia coli* SbmA in the antimicrobial activity of proline-rich peptides. Mol. Microbiol..

[32] Mardirossian M (2018). Fragments of the Nonlytic Proline-Rich Antimicrobial Peptide Bac5 Kill Escherichia coli Cells by Inhibiting Protein Synthesis. Antimicrob. Agents Chemother..

[33] Mardirossian M (2019). Proline-rich peptides with improved antimicrobial activity against *E. coli*, *K. pneumoniae*, and *A. baumannii*. ChemMedChem.

[34] Hartz D, McPheeters DS, Traut R, Gold L (1988). Extension inhibition analysis of translation initiation complexes. Methods Enzymol..

[35] Polikanov YS, Steitz TA, Innis CA (2014). A proton wire to couple aminoacyl-tRNA accommodation and peptide-bond formation on the ribosome. Nat. Struct. Mol. Biol..

[36] Syroegin EA, Aleksandrova EV, Polikanov YS (2022). Structural basis for the inability of chloramphenicol to inhibit peptide bond formation in the presence of A-site glycine. Nucleic Acids Res..

[37] Mangano K (2020). Genome-wide effects of the antimicrobial peptide apidaecin on translation termination in bacteria. eLife.

[38] Mangano, K. et al. Inhibition of translation termination by the antimicrobial peptide drosocin. *Nat. Chem. Biol.*10.1038/s41589-023-01300-x (2023). 10.1038/s41589-023-01300-x PMC10757563 36997647

[39] Lele DS, Talat S, Kaur KJ (2013). The presence of arginine in the Pro-Arg-Pro motif augments the lethality of proline rich antimicrobial peptides of insect source. Int. J. Pept. Res. Ther..

[40] Laurberg M (2008). Structural basis for translation termination on the 70S ribosome. Nature.

[41] Zhou J, Korostelev A, Lancaster L, Noller HF (2012). Crystal structures of 70S ribosomes bound to release factors RF1, RF2 and RF3. Curr. Opin. Struct. Biol..

[42] Dunkle JA, Xiong L, Mankin AS, Cate JH (2010). Structures of the *Escherichia coli* ribosome with antibiotics bound near the peptidyl transferase center explain spectra of drug action. Proc. Natl Acad. Sci. USA.

[43] Svetlov MS, Cohen S, Alsuhebany N, Vazquez-Laslop N, Mankin AS (2020). A long-distance rRNA base pair impacts the ability of macrolide antibiotics to kill bacteria. Proc. Natl Acad. Sci. USA.

[44] van der Stel AX (2021). Structural basis for the tryptophan sensitivity of TnaC-mediated ribosome stalling. Nat. Commun..

[45] Yang K (2017). Structural insights into species-specific features of the ribosome from the human pathogen *Mycobacterium tuberculosis*. Nucleic Acids Res..

[46] Lele DS, Kaur G, Thiruvikraman M, Kaur KJ (2017). Comparing naturally occurring glycosylated forms of proline rich antibacterial peptide, drosocin. Glycoconj. J..

[47] Quan S, Skovgaard O, McLaughlin RE, Buurman ET, Squires CL (2015). Markerless *Escherichia coli*
*rrn* deletion strains for genetic determination of ribosomal binding sites. G3 (Bethesda).

[48] Osterman IA (2020). Tetracenomycin X inhibits translation by binding within the ribosomal exit tunnel. Nat. Chem. Biol..

[49] Kimanius D, Dong L, Sharov G, Nakane T, Scheres SHW (2021). New tools for automated cryo-EM single-particle analysis in RELION-4.0. Biochem. J..

[50] Zheng SQ (2017). MotionCor2: anisotropic correction of beam-induced motion for improved cryo-electron microscopy. Nat. Methods.

[51] Wagner T (2019). SPHIRE-crYOLO is a fast and accurate fully automated particle picker for cryo-EM. Commun. Biol..

[52] Heymann JB (2018). Guidelines for using Bsoft for high resolution reconstruction and validation of biomolecular structures from electron micrographs. Protein Sci..

[53] Watson ZL (2020). Structure of the bacterial ribosome at 2 Å resolution. eLife.

[54] Vagin AA (2004). REFMAC5 dictionary: organization of prior chemical knowledge and guidelines for its use. Acta Crystallogr. D.

[55] Long F (2017). AceDRG: a stereochemical description generator for ligands. Acta Crystallogr. D.

[56] Pettersen EF (2021). UCSF ChimeraX: structure visualization for researchers, educators, and developers. Protein Sci..

[57] Emsley P, Lohkamp B, Scott WG, Cowtan K (2010). Features and development of Coot. Acta Crystallogr. D.

[58] Winn MD (2011). Overview of the CCP4 suite and current developments. Acta Crystallogr. D.

[59] Yamashita K, Palmer CM, Burnley T, Murshudov GN (2021). Cryo-EM single-particle structure refinement and map calculation using Servalcat. Acta Crystallogr. D.

[60] Liebschner D (2019). Macromolecular structure determination using X-rays, neutrons and electrons: recent developments in Phenix. Acta Crystallogr. D.

[61] Chan KH (2020). Mechanism of ribosome rescue by alternative ribosome-rescue factor B. Nat. Commun..

