# Supplementary material for: Structural basis for translation inhibition by the glycosylated drosocin peptide
Source: Nat Chem Biol. 2023 Mar 30;19(9):1072–81. doi: 10.1038/s41589-023-01293-7 (PMC10449632; doi:10.1038/s41589-023-01293-7)
Supplement: Supplementary file 1 — Supplementary Figs. 1–5, Table 1 and References. [file 41589_2023_1293_MOESM1_ESM.pdf]

nature chemical biology

Article https://doi.org/10.1038/s41589-023-01293-7

Structural basis for translation inhibition by the glycosylated drosocin peptide

In the format provided by the authors and unedited

Supplementary Figures

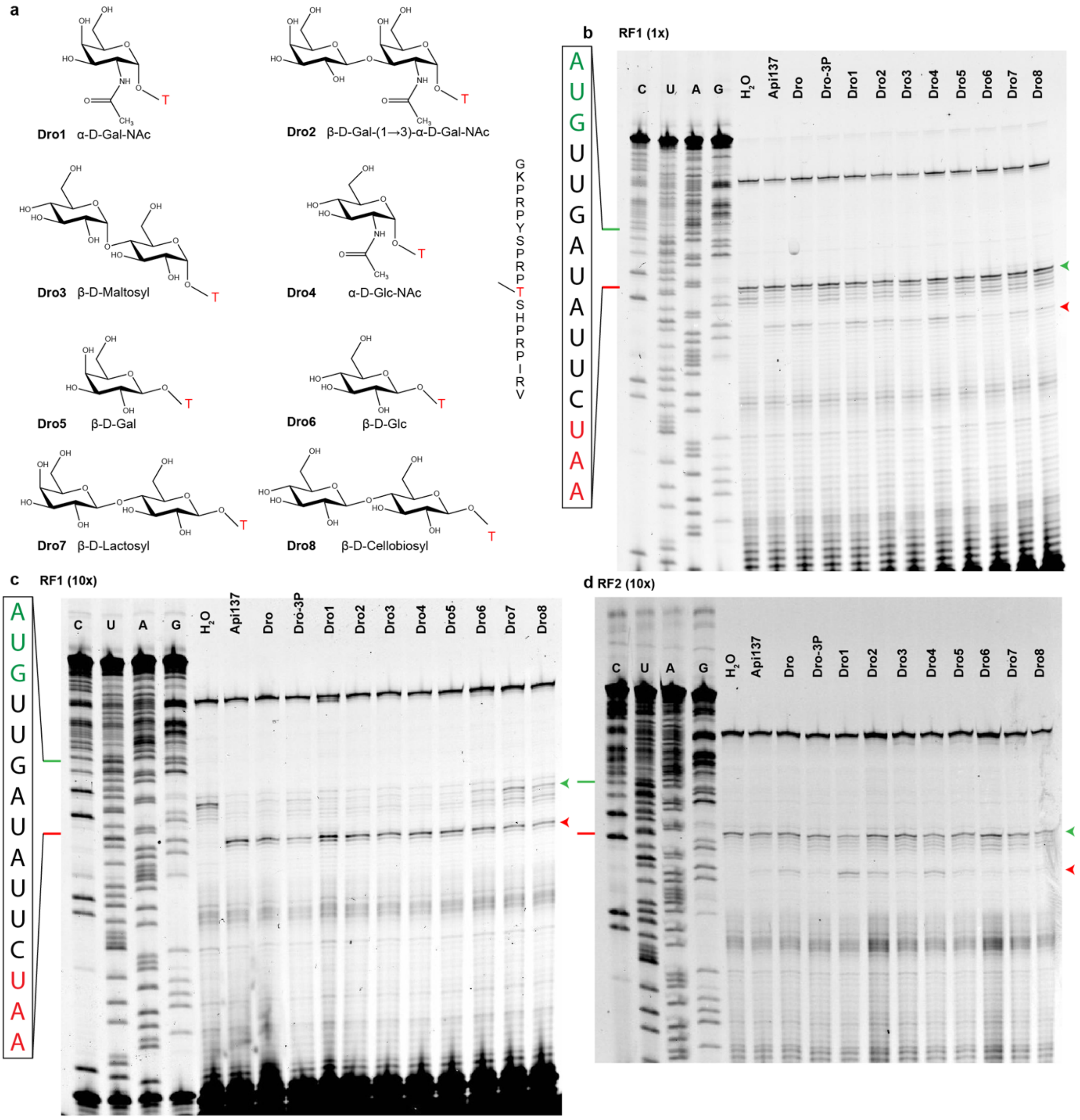

Supplementary Fig. 1: Toeprint of drosocin in presence of release factors. a, Chemical structures of the Thr11 modifications of Dro1-Dro8. **b-d**, Uncropped toeprinting assays from figure 1f-h monitoring the position of ribosomes on an MLIF*-mRNA in the presence of 30 µM Api137 and drosocin derivatives and either (**b**) 1x RF1, (**c**) 10x RF1 or (**d**) 10x RF2. Bands corresponding to ribosomes present at the start and stop codons are indicated by green and red arrows, respectively. The start and stop codons on the sequencing lanes are indicated by green and red lines and correspond to the sequence shown on the left.

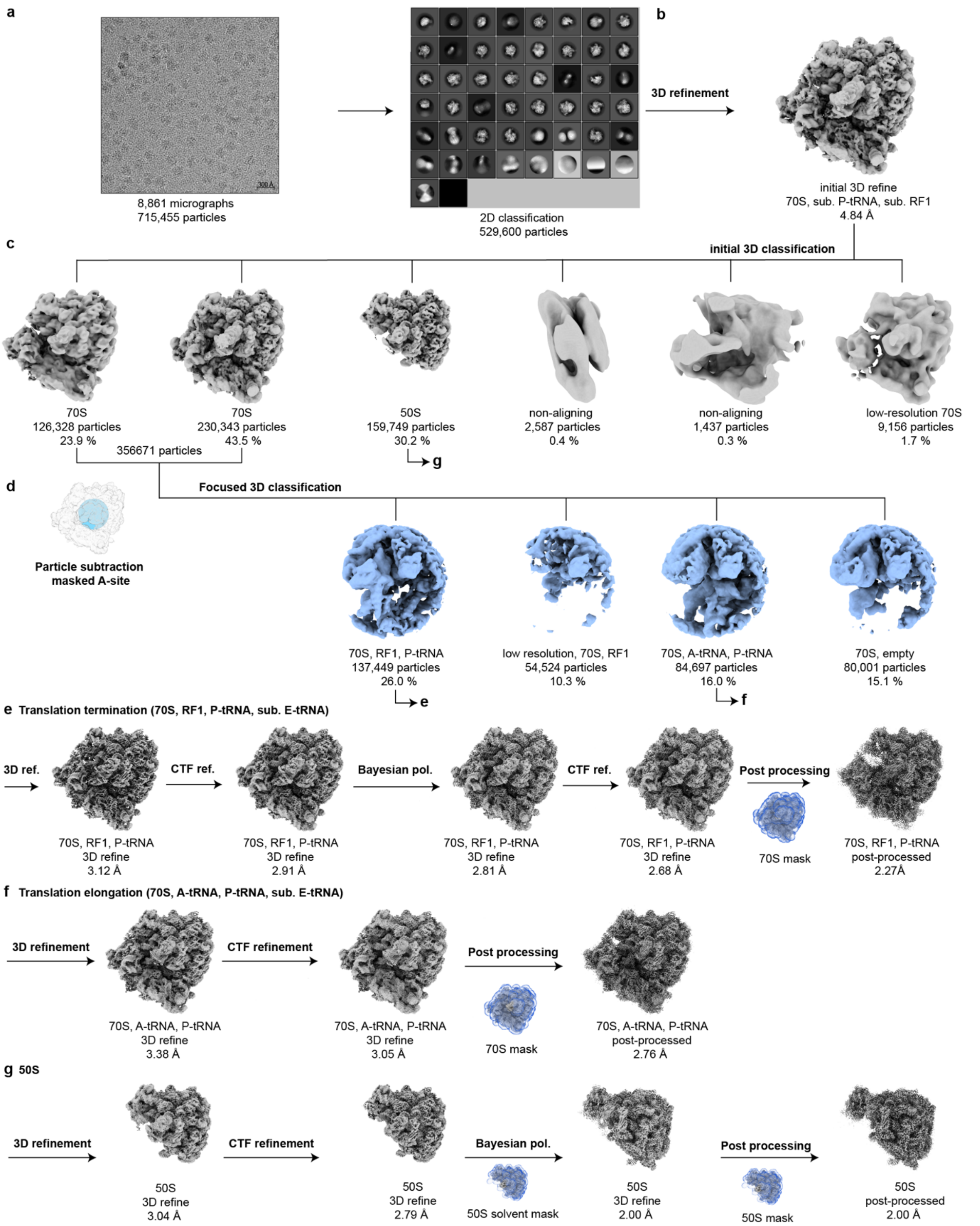

Supplementary Fig. 2: *In silico* sorting scheme. a, From 8,861 micrographs, 715,455 particles were picked and subjected to 2D classification resulting in 529,600 ribosome-like particles. **b**, Particles were initially 3D refined at 3x decimated pixel size. **c**, Initial 3D classification for 70 iterations resulted in six classes. Classes containing 70S were combined and subsorted (356,671 particles) and a class containing 50S (159,749 particles) was further processed. **d**, 70S particles were subtracted with a mask around the A-site and subjected to 150 iterations of focused 3D classification. **e**, Termination complex containing RF1, P-tRNA and sub. E-tRNA density was 3D refined at undecimated pixel size and subjected to CTF refinement (4th order aberrations, beam-tilt, anisotropic magnification and per-particle defocus

value estimation), Bayesian polished and again CTF refined resulting in a final average resolution for the masked reconstruction of 2.3 Å (at $FSC_{0.143}$). **f**, Elongation complex containing A-tRNA and P-tRNA density was 3D refined at undecimated pixel size and subjected to CTF refinement (4th order aberrations, beam-tilt, anisotropic magnification and per-particle defocus value estimation), resulting in a final average resolution for the masked reconstruction of 2.8 Å (at $FSC_{0.143}$). **g**, 50S complex containing A-tRNA and P-tRNA density was 3D refined at undecimated pixel size and subjected to CTF refinement (4th order aberrations, beam-tilt, anisotropic magnification and per-particle defocus value estimation) and Bayesian polishing, resulting in a final average resolution for the masked reconstruction of 2.0 Å (at $FSC_{0.143}$). The single particle analysis was performed a single time and for 3D refinements in RELION, particles are randomly placed in one of two subsets and half-reconstructions are processed independently employing the gold-standard. These subsets are maintained for CTF refinement.

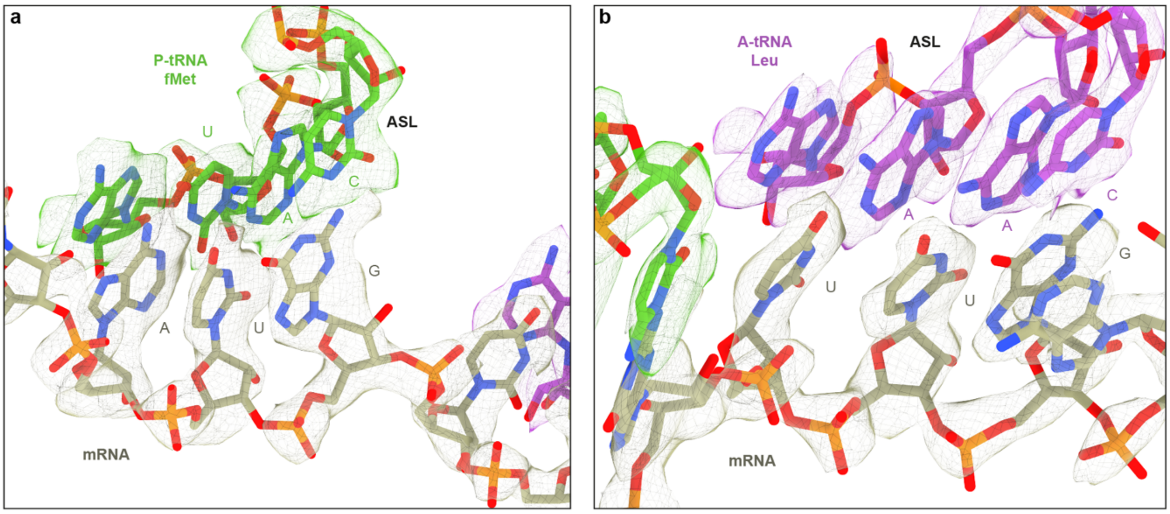

Supplementary Fig. 3: Anticodon stem loop interaction of the elongation complex. a, P-site fMet-tRNA UAC anticodon stem loop (lime) interacting with the AUG start codon of the mRNA (light brown) shown with isolated density (mesh). **b**, A-site Leu-tRNA AAC anticodon stem loop (purple) interacting with the UUG codon of the mRNA (light brown) shown with isolated density (mesh).

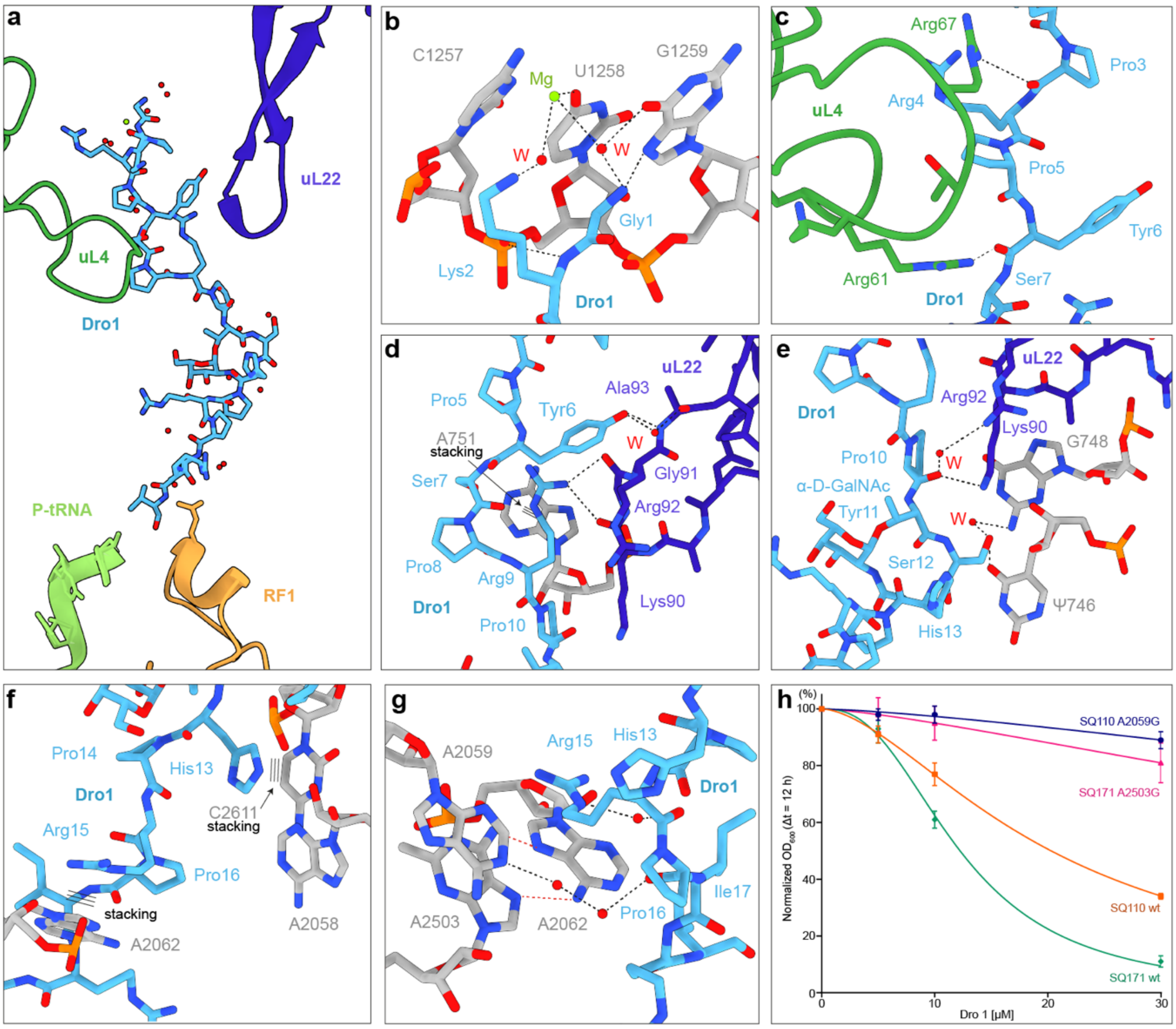

Supplementary Fig. 4 Interactions of drosocin within the exit tunnel. a, The N-terminus of Dro1 (light blue) reaches down the tunnel past the constriction created by the extensions of ribosomal proteins uL4 and uL22. **b,** N-terminal amino group of Dro1 can form potential hydrogen bonding interactions with the N7 of 23S rRNA nucleotide G1259. Additionally, Lys2 establishes two contacts with U1258, one from the backbone amine to the phosphate-oxygen of U1258, and the other mediated via a water molecule between the ε-amino group of the Lys2 sidechain and the O4 of U1258. Removal of first five N-terminal residues (GKPRP), which includes the first PRP motif, completely abolishes activity, suggesting the importance of the N-terminal interactions for drosocin activity, although effects on uptake cannot be excluded[1]. **c-e,** Residues Pro3 to Pro10 of Dro1 are located at the constriction and establish multiple interactions with uL4 and uL22. Specifically, (**c**) the backbone carboxyls of Pro3 and Tyr6 of Dro1 are within hydrogen bonding distance to the sidechains Arg67 and Arg61 of uL4, respectively. (**d**) Interactions with uL22 include hydrogen bonds between the sidechains of Tyr6 and Arg9 of Dro1 with the backbone of Ala93 and Lys90/Gly91 of uL22, respectively. The Tyr6 interaction appears not to be critical since mutation to Phe that lacks the hydroxyl group does not lead to loss of antimicrobial activity[2]. (**e**) The backbone carboxyl of Pro10 of Dro1 can interact with the sidechain of Lys90 of uL22 as well as indirectly with Arg92 via a water molecule. Mutation of Pro10 to Ala abolishes antimicrobial activity[3], presumably by altering the conformation of the peptide within this region. Ser12 of Dro1 can hydrogen bond directly with Ψ746 and form a water-mediated interaction with G748. **h**, *in vivo* inhibitory activity of 5 µM, 10 µM and 30 µM Dro1 on the growth of *E. coli* SQ110 wt (orange), *E. coli* SQ110 A2059G (blue), *E. coli* SQ171 wt (green) and *E. coli* SQ171 A2503G (pink) in LB medium. For each concentration, residual growth values are the OD_{600} at t = 12 h of the treated culture normalized to the untreated one, considered as 100 %. The plotted points represent the mean

for three independent biological replicas, the error bars represent the standard deviation and the technical measurement error of the plate reader. The curves were calculated and plotted by non-linear regression.

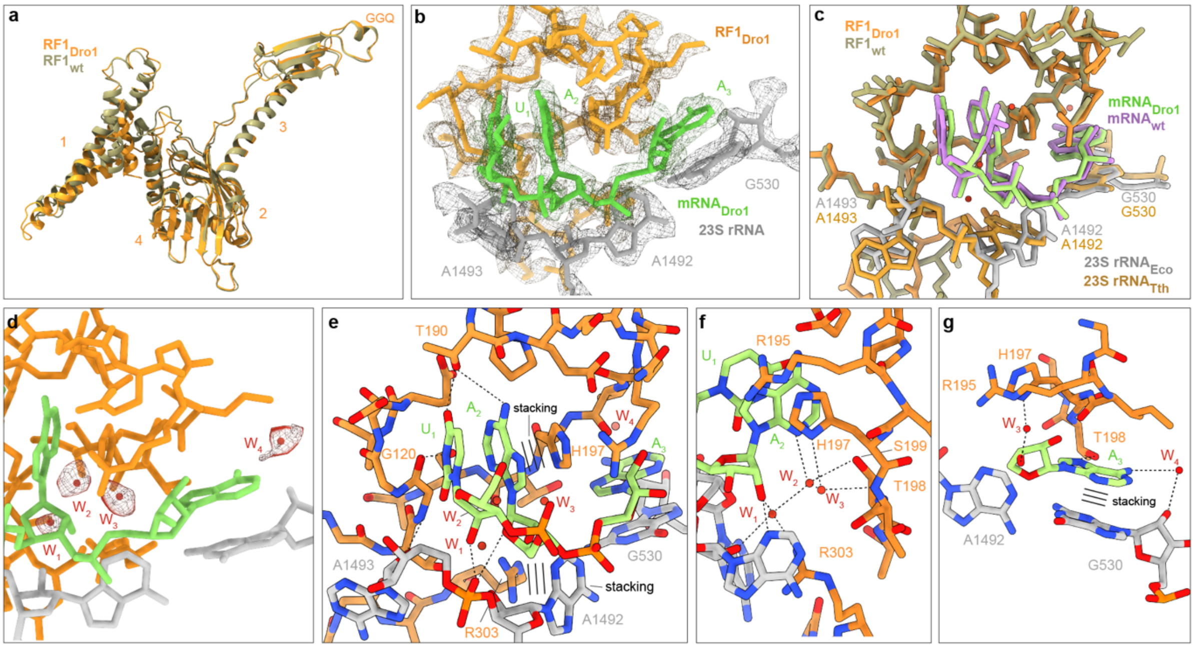

Supplementary Fig. 5: Stop codon recognition in the drosocin-bound termination complex. a, Superimposition of RF1 in the termination complex (orange) with a canonical RF1 (olive, PDB ID 4V63)[4]. **b**, Isolated density (mesh) for the RF1 (orange), UAA stop codon (lime) of the mRNA and 23S rRNA nucleotides (grey). **c**, Superimposition of RF1 (orange), mRNA (lime) and 23S rRNA nucleotides in the termination complex essential for the stop codon recognition with a canonical termination complex RF1 (olive, PDB ID 4V63) [4], mRNA (purple) and 23S rRNA nucleotides (grey). **d**, Isolated density assigned to four additional waters molecules in close proximity to RF1 (orange), mRNA (lime), and 23S rRNA nucleotides (grey) within the drosocin-bound termination complex. **e-f**, Potential water-mediated and direct interactions of the UAA nucleotides of the stop codon (lime) with side chains G120, T190, H197, T198, S199 and R303 of RF1 (orange) as well as A1492, A1493 and G530 nucleotides of the 23S rRNA (grey). Hydrogen bonds are indicated by dashed lines and stacking by three lines.

Table S1. Cryo-EM data collection, modelling and refinement statistics.

	**Termination complex** (EMD-15488) (PDB 8AKN)	**Elongation complex** (EMD-15523) (PDB 8AM9)	**50S Complex** (EMD-15533) (PDB 8ANA)
**Data collection**			
Magnification (×)	96,000	96,000	96,000
Voltage (kV)	300	300	300
Electron exposure ($e^{-}/Å^2$)	40	40	40
Defocus range (μm)	-0.4 to -0.9	-0.4 to -0.9	-0.4 to -0.9
Pixel size (Å)	0.80	0.80	0.80
Symmetry imposed	C1	C1	C1
Initial particle images (no.)	529,600	529,600	529,600
Final particle images (no.)	137,449	84,697	159,749
Map resolution (Å)	2.3	2.8	2.0
FSC threshold	0.143	0.143	0.143
Map resolution range (Å)	1.9-3.5	2.0-5.0	1.8-3.5
**Refinement**			
Initial model used (PDB)	7K00	7K00	7K00
Model resolution (Å)	2.7	3.1	2.5
FSC threshold	0.5	0.5	0.5
Map CC around atoms	0.80	0.81	0.85
Map CC whole unit cell	0.79	0.78	0.83
Map sharpening B factor ($Å^2$)	-4.7	-26.7	-7.2
Model composition			
Non-hydrogen atoms	146,514	142,038	86,889
Protein residues	5,960	5,606	3,196
RNA bases	4,552	4,554	2,872
B factors ($Å^2$)			
Protein	81.3	12.7	60.6
Nucleotide	67.7	15.9	49.2
R.M.S. deviations			
Bond lengths (Å)	0.014	0.008	0.012
Bond angles (°)	1.783	1.014	1.552
Validation			
MolProbity score	1.82	2.01	1.36
Clash score	3.24	5.74	1.75
Poor rotamers (%)	2.19	3.55	1.00
Ramachandran statistics			
Favored (%)	93.17	95.95	93.48
Allowed (%)	5.95	3.80	5.75
Disallowed (%)	0.89	0.25	0.77

Supplementary References

1. Bulet, P., Urge, L., Ohresser, S., Hetru, C., and Otvos, L., Jr. (1996). Enlarged scale chemical synthesis and range of activity of drosocin, an O-glycosylated antibacterial peptide of Drosophila. Eur J Biochem *238*, 64-69. 10.1111/j.1432-1033.1996.0064q.x.
2. de Visser, P.C., van Hooft, P.A., de Vries, A.M., de Jong, A., van der Marel, G.A., Overkleeft, H.S., and Noort, D. (2005). Biological evaluation of Tyr6 and Ser7 modified drosocin analogues. Bioorg Med Chem Lett *15*, 2902-2905. 10.1016/j.bmcl.2005.03.074.
3. Ahn, M., Sohn, H., Nan, Y.H., Murugan, R.N., Cheong, C., Eun Kyoung Ryu, Kim, E.-H., Kang, S.W., Kim, E.J., Shin, S.Y., and Bang, J.K. (2011). Functional and Structural Characterization of Drosocin and its Derivatives Linked O-GalNAc at Thr11 Residue. Bull. Korean Chem. Soc. *32*, 3327-3332.
4. Laurberg, M., Asahara, H., Korostelev, A., Zhu, J., Trakhanov, S., and Noller, H.F. (2008). Structural basis for translation termination on the 70S ribosome. Nature *454*, 852-857.
